# Microbial Community Composition and Function in Jiangsu Oil Reservoir Cores, China

**DOI:** 10.1111/1758-2229.70229

**Published:** 2025-11-26

**Authors:** Bo‐Wen Wang, Yi‐Fan Liu, Le‐Gang Chen, Biao Wang, Zhi‐Hong Qian, Fan Yang, Jia‐Cheng Cai, Lei Zhou, Shi‐Zhong Yang, Ji‐Dong Gu, Bo‐Zhong Mu

**Affiliations:** ^1^ State Key Laboratory of Bioreactor Engineering and School of Chemistry and Molecular Engineering East China University of Science and Technology Shanghai PR China; ^2^ Engineering Research Center of MEOR East China University of Science and Technology Shanghai PR China; ^3^ Research Institute of Petroleum Engineering Jiangsu Oilfield, Sinopec Yangzhou Jiangsu PR China; ^4^ College of Chemistry and Molecular Engineering East China University of Science and Technology Shanghai PR China; ^5^ Environmental Science and Engineering Group Guangdong Technion Israel Institute of Technology Shantou Guangdong PR China

**Keywords:** hydrocarbon degradation, microbial composition, shale core, shale oil reservoir

## Abstract

Shale oil reservoirs are typically characterised by elevated temperatures, confined spaces and oligotrophic conditions. Understanding the role of microorganisms in shale oil reservoirs is essential for elucidating biogeochemical cycles and the origins of life. However, the composition and metabolic functions of microbial communities in shale oil reservoirs remain elusive. In this study, shale core samples were collected from the HY1‐1 and HY7 wells in the Jiangsu Oilfield. A combination of X‐ray fluorescence, powder X‐ray diffraction and scanning electron microscope analyses revealed that the samples contained various transition metals, abundant clay minerals and numerous pores with diameters greater than 1 μm. Fractionation of extracted crude oil fractions revealed that HY1‐1 and HY7 contained 60% and 74% saturated hydrocarbons, primarily comprising C_11_–C_35_ *n*‐alkanes. Various hydrocarbon‐degrading microorganisms, including *Marinobacter*, *Alcanivorax*, *Alkanindiges* and *Nocardioides* were present in HY1‐1 or HY7 samples. Metagenomic analysis showed the presence of genes associated with aerobic hydrocarbon degradation, denitrification and DNRA in the HY7 sample, suggesting that microorganisms may utilise crude oil for growth and participate in the subsurface carbon and nitrogen cycle. This study elucidates the microbial community structure and functional gene profiles in shale core samples, providing critical insights for harnessing in situ microorganisms in shale oil reservoir development.

## Graphical abstract

Shale oil reservoirs meet the basic requirements for microbial growth. Microorganisms, crude oil and water are distributed within the pore spaces of the reservoir formation. In situ microorganisms utilise crude oil for growth and reproduction.

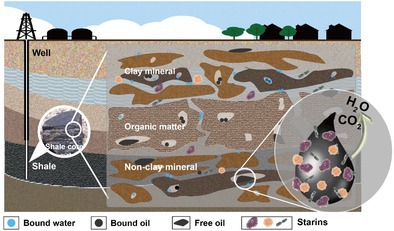

## 1 Introduction

Shale oil is a significant unconventional petroleum resource. The U.S. Energy Information Administration (EIA) estimated that global shale oil reserves amount to approximately 419 billion barrels (Stolz et al. 2022), accounting for 20%–50% of the world's total oil reserves (Zou et al. 2013). Continental shale oil reservoirs in China are generally buried at depths of 2400–3000 m (Xiang et al. 2015), where organic‐rich shale is interbedded with other lithological rock layers (Zou et al. 2013). The abnormally high pressure in these reservoirs results in limited storage space, low porosity and ultra‐low permeability (Jiang et al. 2016). The complex origin of organic matter and deposition processes results in the high heterogeneity of shale reservoirs (Sharma et al. 2017). Shale oil was formed within source rocks and occurs in adsorbed or free states within micron‐ or nano‐scale pores and fractures. It typically exhibits a high degree of thermal maturity and relatively light oil composition (Xiang et al. 2015; Wang et al. 2019). Shale oil reservoirs, as subsurface environments, pose significant challenges to microbial survival due to elevated temperatures, high pressures, oligotrophic conditions and extremely limited pore space (Wentzel et al. 2013).

Considering the harsh physicochemical conditions of shale reservoirs, the existence of indigenous microbiomes in subsurface shale was still under debate (Tinker et al. 2020). Research on microorganisms inhabiting extreme environments analogous to shale oil reservoirs, such as deep‐sea hydrothermal vents, subsurface and hypersaline habitats, has demonstrated that these organisms have developed diverse strategies to adapt to environmental pressures (Shu and Huang 2022). Microorganisms adapted to high‐temperature environments by enhancing the stability of their cell membranes, proteins and DNA (Shu and Huang 2022). In oligotrophic conditions, microbial metabolic rates were 10^4^ to 10^6^‐fold lower than in nutrient‐rich environments, accompanied by prolonged generation times (Hoehler and Jørgensen 2013) and reduced genome sizes (Wu et al. 2015). Among all factors limiting microbial life in natural shale environments, spatial constraints were considered the most critical (Mouser et al. 2016). Microorganisms were even capable of surviving in micropores as small as 0.2 μm in diameter (Mouser et al. 2016). Shale oil reservoirs were widely distributed with pore throats ranging from 50 to 300 nm, and localised development of micro‐scale pores may also occur (Zou et al. 2013). Therefore, shale oil reservoirs exhibited potential for microbial survival. Due to the challenges associated with sampling and handling core samples, analysing liquid samples from shale reservoirs, such as produced water and hydraulic fracturing flowback fluid as a proxy, provided an alternative approach to studying the composition and function of microbial communities. Previous cultivation experiments utilising produced water as the inoculum source, coupled with metagenomic analysis of the original samples, have demonstrated that the microbial communities present in the produced water or flowback fluid possess functions associated with hydrocarbon degradation, nitrogen cycling, sulphur cycling and the degradation of surfactants and complex polymers (An et al. 2017; Evans et al. 2019; Amundson et al. 2022). However, the introduction of numerous organic and inorganic chemical additives carried by fracturing fluids during hydraulic fracturing (Mouser et al. 2016). It may also provide biological and chemical inputs that facilitate microbial colonisation and sustained growth in deep terrestrial subsurface environments (Daly et al. 2016). Produced water extracted from shale reservoirs comprises a mixture of fracturing fluids and formation water. Consequently, some researchers contend that the microbial communities detected in this fluid may not accurately reflect the native microbiota of the reservoir itself (Akob et al. 2015). Therefore, core samples were identified as the most effective approach for understanding the composition and functions of microbial communities in shale oil reservoirs.

In shale oil reservoir formation, due to high temperatures, limited space and near‐equilibrium redox conditions at the microscale, microbial communities were sparse and almost entirely in a dormant state (Buchwalter et al. 2015). In deep subsurface pristine shale, the estimated cell count is between 10^1^–10^5^ cells g^−1^, and the phospholipid fatty acid concentration is < 32 pmol g^−1^ (Onstott et al. 1998). Krumholz et al. (1997) confirmed the existence of a small microbial community within the shale core, utilising the organic matter present in the shale as their energy source. Tucker et al. (2015) compared the community composition of core samples, injection water and produced water from the Marcellus shale, concluding that methanogenic archaea in the produced water originated from the core rather than the injection water. Additionally, Trexler identified phospholipid fatty acid components extracted from the core samples that were absent in the drilling mud (Trexler 2017). Subsequent studies indicated the potential presence of extremophiles in Marcellus shale, such as *Deinococcus radiodurans* , known for their radiation resistance (Tucker and Mroz 2018). In shale systems, locally deposited organic matter was likely the primary electron donor source for deep microbial activity (Yin et al. 2016). Studies have shown that indigenous microorganisms at the deep shale and shallower shale‐sandstone layers can survive in micro‐pores, larger fractures and rock interfaces rich in hydrocarbons by fermentation, iron cycling and sulphur cycling metabolism (Cluff et al. 2014). Hernandez‐Becerra et al. (2023) analysed produced water samples treated with sterilised injection water and identified abundant hydrocarbon‐degrading bacteria, including *Shewanellaceae*, *Marinobacteraceae*, *Halomonadaceae* and *Pseudomonadaceae*. A large number of hydrocarbon‐degrading microorganisms were identified in shale oil reservoir core samples (Ridley and Voordouw 2018; Tsesmetzis et al. 2018; Schlegel et al. 2013). The metagenomic analysis results confirmed that microorganisms in shale fractures have the ability to ferment organic additives (Daly et al. 2016). Methane is the ultimate product of organic matter degradation in environments with limited electron acceptor availability. Numerous studies have consistently demonstrated the ubiquitous distribution of methanogenic archaea within shale oil reservoirs (Amundson et al. 2022; Daly et al. 2016; Tucker et al. 2015; Booker et al. 2017). Meanwhile, sulphate‐reducing bacteria (Fredrickson et al. 1997), acetate‐producing bacteria (Krumholz et al. 1997), and iron‐reducing bacteria (Colwell et al. 1997) were cultured from original shale core samples. However, most of the reported core samples were from hotspots such as the Marcellus shale, and there was still a lack of sufficient research on the microbial community composition and functions in the shale oil reservoirs of China.

In this study, shale core samples were collected from two shale oil wells, HY1‐1 and HY7, in the Jiangsu Oilfield. First, core sample lithology was characterised using X‐ray fluorescence (XRF), powder X‐ray diffraction (PXRD) and scanning electron microscope (SEM). Next, gas chromatography–mass spectrometry (GC–MS) was employed to analyse saturated and aromatic hydrocarbon compositions. Key geochemical parameters, such as the pristane/phytane ratio, OEP, CPI and sterane isomer ratios C_29_‐ββ/(ββ + αα) and C_29_‐ααα20S/(20S + 20R), were evaluated to assess crude oil geochemistry characteristics. CLSM and 16S rRNA amplicon sequencing were then used to determine the spatial distribution of viable cells and microbial community composition, respectively. Finally, metagenomic analysis elucidated the metabolic potential of these microbial communities. This study provides a case for understanding the microbial communities in China's deep‐subsurface shale oil reservoirs and a theoretical reference for the activation and utilisation of in situ microbial resources.

## 2 Materials and Methods

### 2.1 Sample Description

**FIGURE 1:**
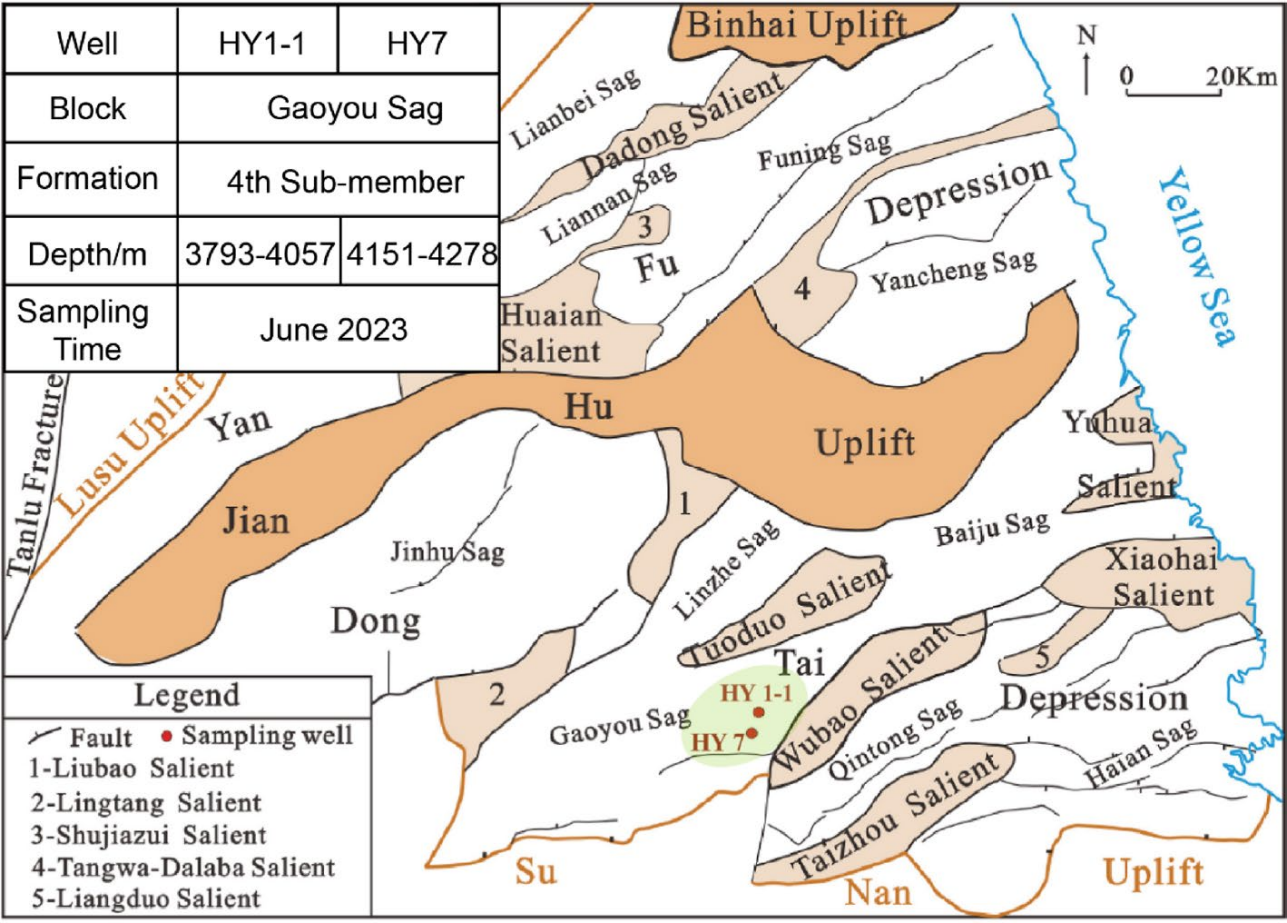
Map of the study area showing locations of the sampling wells.

Shale core fragment samples were obtained from Jiangsu Oilfield Engineering Institute in June 2023; both core fragments were broken off from the cylindrical shale core. Cylindrical shale core samples were collected from the HY1‐1 and HY7 shale oil wells in the Gaoyou Sag of the Jiangsu Oilfield (Figure 1). After detaching, the fragments were sealed in bags and stored at room temperature. The core column samples were collected from exploration wells, which were drilled in May and August of 2022, respectively. Subsequently, the HY7 well underwent carbon dioxide fracturing and production in June 2023, while the HY1‐1 well served as a control and did not undergo carbon dioxide fracturing. After the core fragment samples were collected, they were frozen with dry ice for transportation to the laboratory and then stored at −20°C until further processing. To remove any exogenous microorganisms on the sample surface, we trimmed away the outer layer of the rock. Ethanol was used for sterilisation during the trimming process to prevent new microbial contamination. After the procedure, the samples were immediately sealed in sterile bags and stored at 4°C until further processing. The trimmed inner rock (TIR) was prepared into powders and small pieces under controlled conditions to prevent contamination and was subsequently used for DNA extraction and CLSM observation, respectively. The trimmed outer rock (TOR) was prepared into powders and pieces. TOR powder was used for PXRD detection and organic matter extraction. TOR pieces were used for XRF and SEM observation, respectively. Details of the operations were provided in the Supporting Information.

### 2.2 Analysis of Elemental and Mineral Composition and Observation of Pore Size Distribution

Element distribution and relative abundance were detected using an XRF on a BRUKER M4 TORNADO with Rh Rh‐targeted X‐ray tube and double‐sided silicon drift detector. SEM on a ZEISS Sigma 360 with an InLens‐SE2‐detector was used to characterise the surface morphology of the shale rock, and the acceleration voltage was 10 kV. Primary mineral composition was determined using a PXRD (Rigaku SmartLab with a 9 kW rotating anode, corresponding operation voltage and current at 40 kV and 100 mA, respectively). The diameter of the micropores on the sample surface was measured through nano‐measurer software (http://nano‐measurer.software.informer.com/).

### 2.3 Characterisation of Shale Oil Family Composition

The family composition of shale oil was extracted based on the Chinese Standard Petroleum Test Method SY/T 5119‐2016. In brief, Soluble organic matter from the powder was extracted using *n*‐hexane. The filtrate was separated using a silica/alumina chromatography column and eluted sequentially with *n*‐hexane, a mixture of *n*‐hexane: dichloromethane (= 2:1 *Vol*), and a mixture of chloroform: methanol (= 1:1 *Vol*). This procedure yielded solutions containing saturated hydrocarbons, aromatic hydrocarbons and resins. The solvent in each fraction was evaporated to a constant weight, and the fractions were individually weighed. The mass fraction of each component ($w_{i}$) was then calculated using Equation (1).

(1) $$w_{i}=\frac{m_{i}}{m_{t}}\times 100\%$$

where $m_{i}$ and $m_{t}$ represent the mass of component *i* and the total mass of the family composition, respectively.

Detailed composition of saturated hydrocarbons and aromatic hydrocarbons was assessed by GC–MS conducted using Agilent·8890‐5977C and Agilent 7890A‐5975C, respectively. Both instruments were equipped with an HP‐5MS elastic quartz capillary column (60 m × 0.25 mm × 0.25 μm). Helium was used as the carrier gas at a constant flow rate of 1 mL/min. The ion source was operated in the electron ionisation mode with an electron beam energy of 70 eV. The mass spectrometer was operated in both full‐scan and selected ion monitoring modes following calibration with a standard reference library.

### 2.4 Observation of the Distribution of Live and Dead Microbes on Shale Surfaces

Bacterial Viability Kits (L7012, Thermo Fisher Scientific, USA) were used for staining microorganisms. The fluorescent stains SYTO 9 and PI were diluted in ultra‐pure water to give final concentrations of 0.167 and 0.8 mM, respectively. A 10 μL aliquot of the diluted staining solution was applied to the sample surfaces and allowed to incubate for approximately 5 min. Subsequently, the samples were rinsed with sterile water to remove any unbound dye. After removing moisture from the surface, the sample was observed using a CLSM on a ZEISS LSM 900 Mat. Fluorescence imaging of SYTO nine‐stained cells was performed using an excitation wavelength of 488 nm and an emission wavelength of 525 nm. For PI‐stained cells, fluorescence imaging was carried out with an excitation wavelength of 561 nm and an emission wavelength of 595 nm. The raw images were processed using ZEISS Zen Lite software.

### 2.5 DNA Extraction

DNA was extracted from TIR powder samples using the magnetic bead‐based soil DNA extraction kit (DC306‐09, FINDROP, China) following the manufacturer's protocol.

### 2.6 16S rRNA Gene Sequencing and Metagenomic Analysis

A total of 250 bp paired‐end reads were generated by subjecting all 16S rRNA gene libraries to sequencing on the Illumina HiSeq 2500 platform. The raw reads of 16S rRNA gene amplicons were quality‐filtered by fastp. The filtered data were denoised, trimmed, merged and chimaeras removed using DADA2 package in R, and the amplicon sequence variants were assigned taxonomic information using the Silva 138 database.

Metagenomic raw reads were quality‐filtered using Trim_galore and adapters were trimmed utilising Cutadapt. By using the SPAdes (v3.13.2) in meta mode, we assembled the short reads into scaffolds. The analysis of the short‐reads was performed by using Bowtie2 (v2.5.4) in paired‐end mode with the –S option to obtain the output in SAM format. The abundance of each gene was calculated, represented by transcripts per million values (TPM) using eXpress (v1.5.1). The coding sequences of the scaffolds were predicted using the Prodigal programme (Hyatt et al. 2010). Then the protein sequences file was submitted to the KEGG database for functional annotation using the GhostKOALA tool. The visualisation of functional gene abundance was completed with R package ‘ggplot2’.

## 3 Results

### 3.1 Petrology and Shale Lithofacies

**FIGURE 2:**
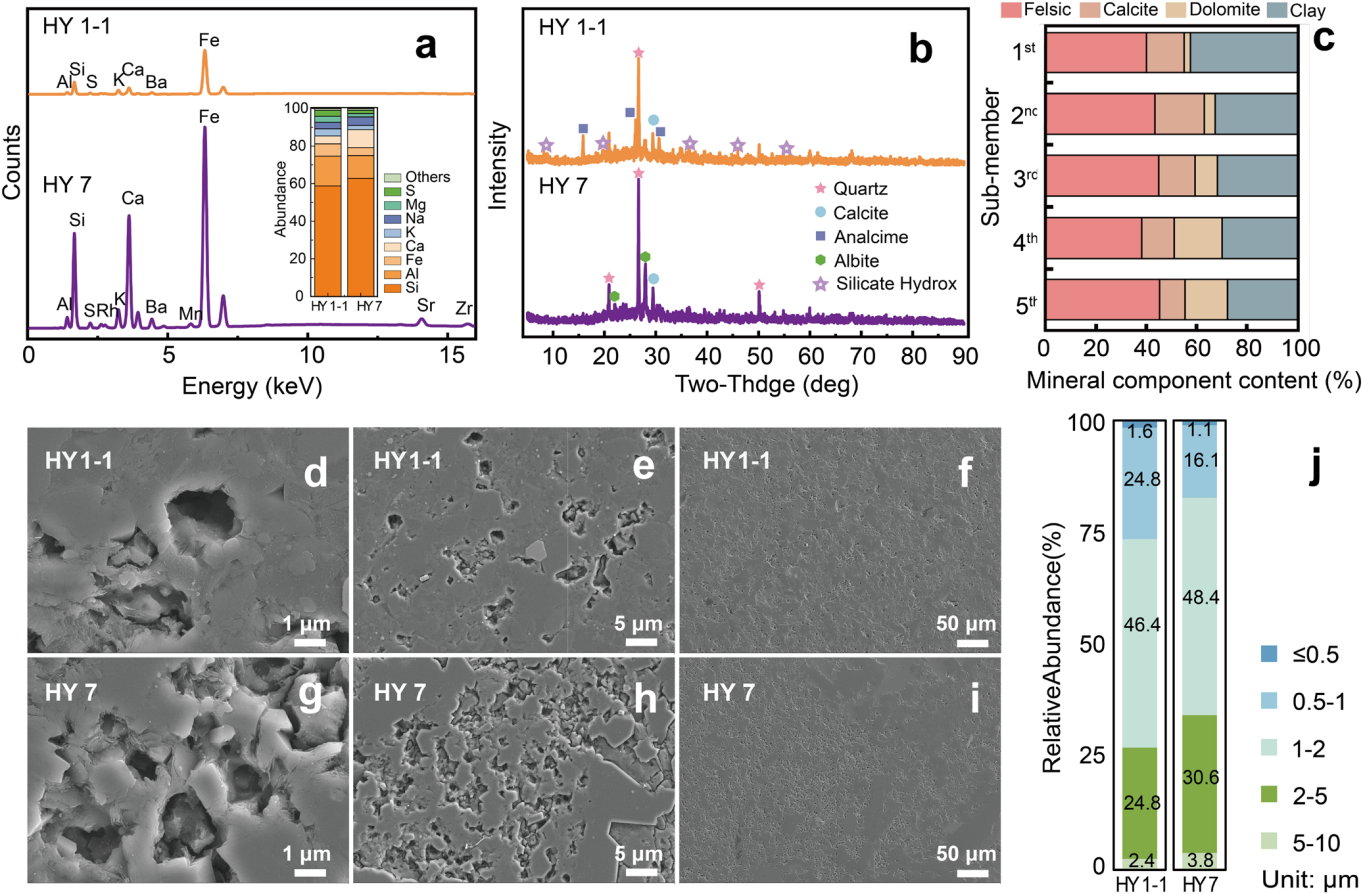
Petrology and shale lithofacies characterisation. X‐ray Fluorescence spectra showing the major elements in shale cores and elemental concentration (a); Lithic facies identification result of the X‐ray diffraction pattern in the shale core (b) and the main mineral content in the Funing formation (c) (Duan et al. 2024); SEM images of HY1‐1 (d–f) and HY7 (g–i) at different resolutions and the relative abundance of micropores with different pore sizes (j).

The second member of the Funing Formation in the Gaoyou Sag (Subei Basin) comprises a lacustrine clastic sedimentary system with associated chemical carbonate deposits, forming an extensive succession of dark‐grey mudstone, mud shale and marl (Luo and Xing 2023). The XRF spectra of the main elements of the study analytes and elemental composition (Figure 2a) demonstrated that the elemental composition of the analytes was quite similar, with silicon being the most abundant element, accounting for approximately 60%. Aluminium was the most prevalent metallic element, and there was also a significant presence of iron and sulphur. Table S1 lists the mass fractions of all elements in the analytes as detected by XRF. It can be observed that there was a considerable amount of trace transition metals present, and the distribution of all elements within the sample was relatively uniform (Figure S1).

The bulk mineral composition of core samples from wells HY1‐1 and HY7 was analysed using PXRD. The analysis revealed quartz as the predominant mineral phase, with calcite present in both samples. Analcime and silicate hydroxide (both silicate minerals) were detected exclusively in core HY1‐1, whereas albite occurred solely in core HY7.

The surface of the analytes was observed using a SEM, with the resulting images shown in Figure 2d–i. The HY1‐1 core surface displayed relatively uniform pore distribution, contrasting with HY7, which showed extensive pore‐free regions (Figure 2i). In both analytes, more than 70% of the micropores have diameters greater than 1 μm. Approximately 50% of the micropores have diameters ranging from 1 to 2 μm, while less than 2% of the micropores have diameters smaller than 0.5 μm (Figure 2j).

### 3.2 Characteristics of Crude Oil Family Compositions

**FIGURE 3:**
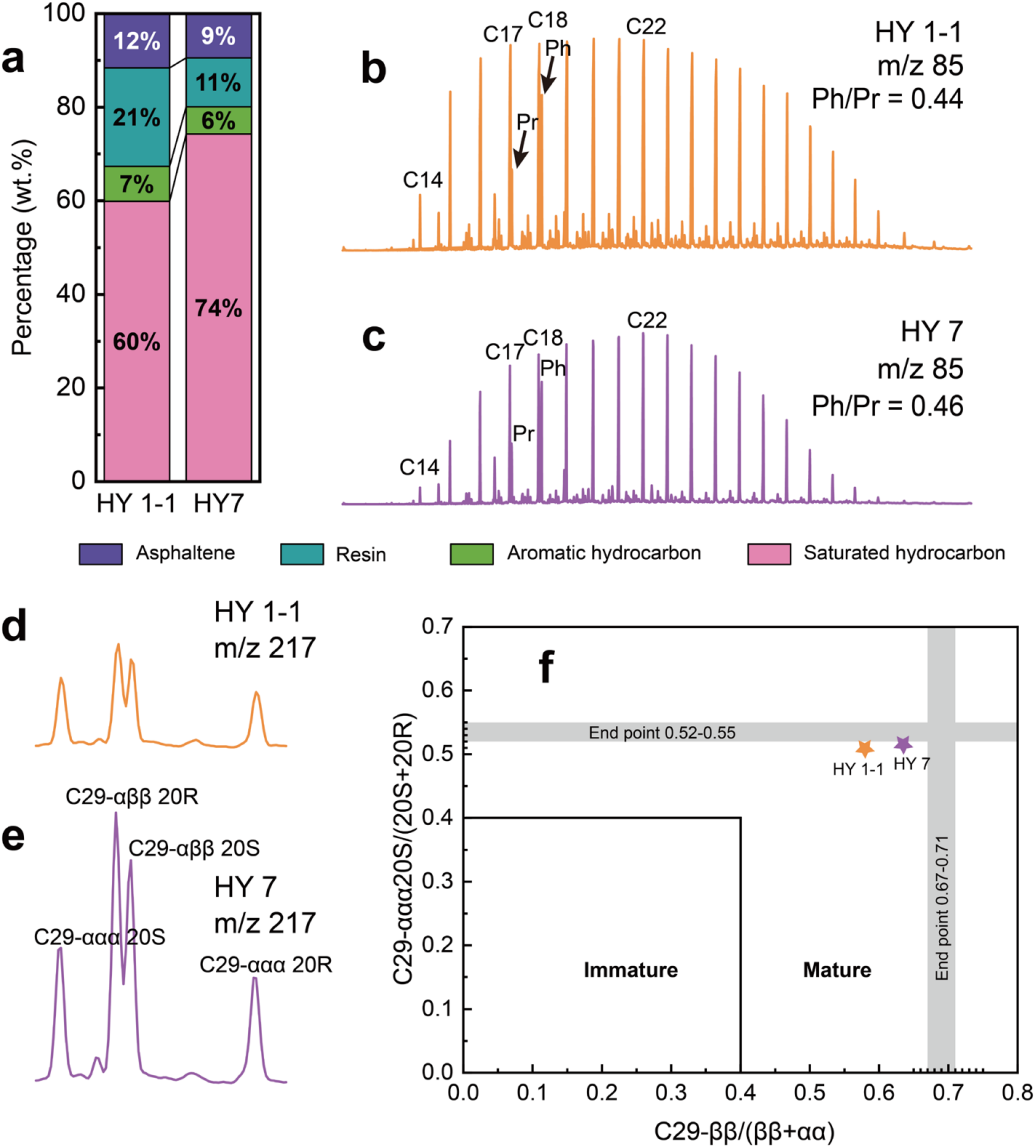
Component of extracted crude oil from shale core samples. Mass fraction of family compositions in shale oil (a); Extract ion chromatogram (EIC) of saturated hydrocarbon of *m*/*z* 85 (b, c) and 217 (d, e); Cross‐plots of sterane index of C29‐ββ/(ββ + αα) and C29‐ααα20S/(20S + 20R) ratios (f).

In the HY1‐1 and HY7 shale cores, the mass fractions of crude oil family compositions account for 0.10% and 0.07% of the total sample mass (Table S2), respectively. Meanwhile, the main component was saturated hydrocarbons (60% in HY1‐1 and 74% in HY7), followed by resins, asphaltenes and aromatics (Table S3, Figure 3a).

The pristane‐to‐phytane ratio (Pr/Ph) in crude oil serves as a redox proxy for reconstructing paleo‐depositional conditions. The Pr/Ph ratios in both HY1‐1 and HY7 were less than 0.5, indicating that the sedimentary sequence was deposited under anoxic paleoenvironmental conditions (Didyk et al. 1978). The *n*‐alkane carbon number distribution in both samples ranges broadly from C_11_ to C_35_ (Figure 3b,c), the OEP values (0.95 and 0.96) and CPI values (0.99 and 1) were approximating unity. The analysis based on the cross‐plots of sterane index of C_29_‐ββ/(ββ + αα) and C_29_ααα‐20S/(20S + 20R) ratios (Figure 3f) further confirms the characteristics of mature oil (Peters and Moldowan 1992). The characteristic symmetric ‘V’‐shaped distribution pattern of C_27_–C_29_ regular steranes (Table S4, Figure S2) suggests mixed contributions from both aquatic organisms and higher plants, with minimal dominance of aquatic sources (Wen‐Yen and Meinschein 1976; Cheng et al. 2019). The predominance of high molecular weight (Mw) *n‐*alkanes (≥C_27_) reflects terrigenous higher plant contributions, whereas the mid Mw homologues (C_21_–C_25_) suggest freshwater aquatic origins (Chevalier et al. 2015). The dominance of mid Mw *n‐*alkanes, along with detectable high Mw constituents, corroborates the regular steranes distribution pattern.

### 3.3 Composition and Function of the Microbial Community in Shale Cores

**FIGURE 4:**
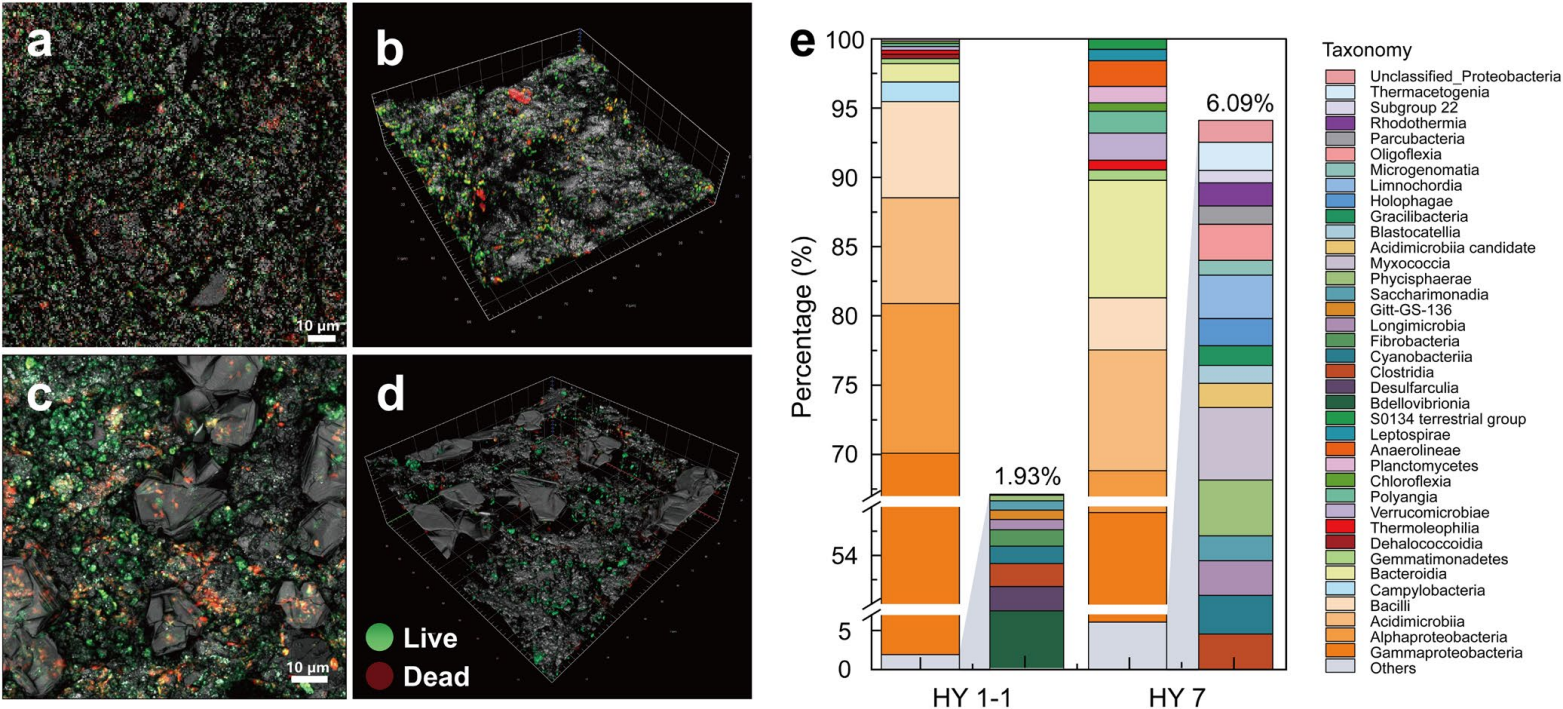
CLSM fluorescence images shale rock sample surface and reconstructed by 3D scanning of HY1‐1 (a, b) and HY7 (c, d); Relative abundance of major taxonomic groups at the class level (e). A C Epiplan‐Apochromat 20×/0.7 DlC objective lens was used with the following settings: Pinhole diameter of 5.00 AU (169 μm), frame scan mode, pixel dwell time of 0.7 μs, frame acquisition time of 467.17 ms, LSM scan speed setting of 9, and an effective numerical aperture (NA) of 0.7.

The fluorescence imaging results of the shale core surface (Figure 4a–d) showed that the surfaces of HY1‐1 and HY7 contain a high number of live and dead microorganisms. These microorganisms predominantly accumulate in localised depressions, while their presence on the elevated and relatively smooth rock surfaces was minimal.

At the class level (Figure 4e), the bacterial community composition of the two samples was quite similar, with *Gammaproteobacteria*, *Alphaproteobacteria*, *Acidimicrobiia* and *Bacilli* comprising over 70% of the total abundances. Additionally, the HY7 sample contained 8.5% *Bacteroidota*, whereas its abundance in HY1‐1 was only 1.3%. At the genus level (Tables S5 and S6), 230 and 161 genera were detected in HY1‐1 and HY7, respectively. In HY1‐1, the most abundant genera were *Pseudomonas*, *Unclassified_Enterobacteriaceae*, *Acinetobacter*, *Unclassified_Planococcaceae* and *Comamonas*, with relative abundances of 35.29%, 6.81%, 3.51%, 2.10% and 1.97%, respectively. On the other hand, in HY7, the dominant genera included *Burkholderia‐Caballeronia‐Paraburkholderia*, *Brevundimonas*, *Unclassified_Microbacteriaceae*, *Pseudoxanthomonas* and *Pseudomonas*, which exhibited relative abundances of 30.49%, 2.68%, 1.98%, 1.96% and 1.90%, respectively.

The analysis of the archaeal community composition revealed that *Methanobrevibacter* was the sole archaeon detected in the HY1‐1 sample, while no archaea were identified in HY7.

**FIGURE 5:**
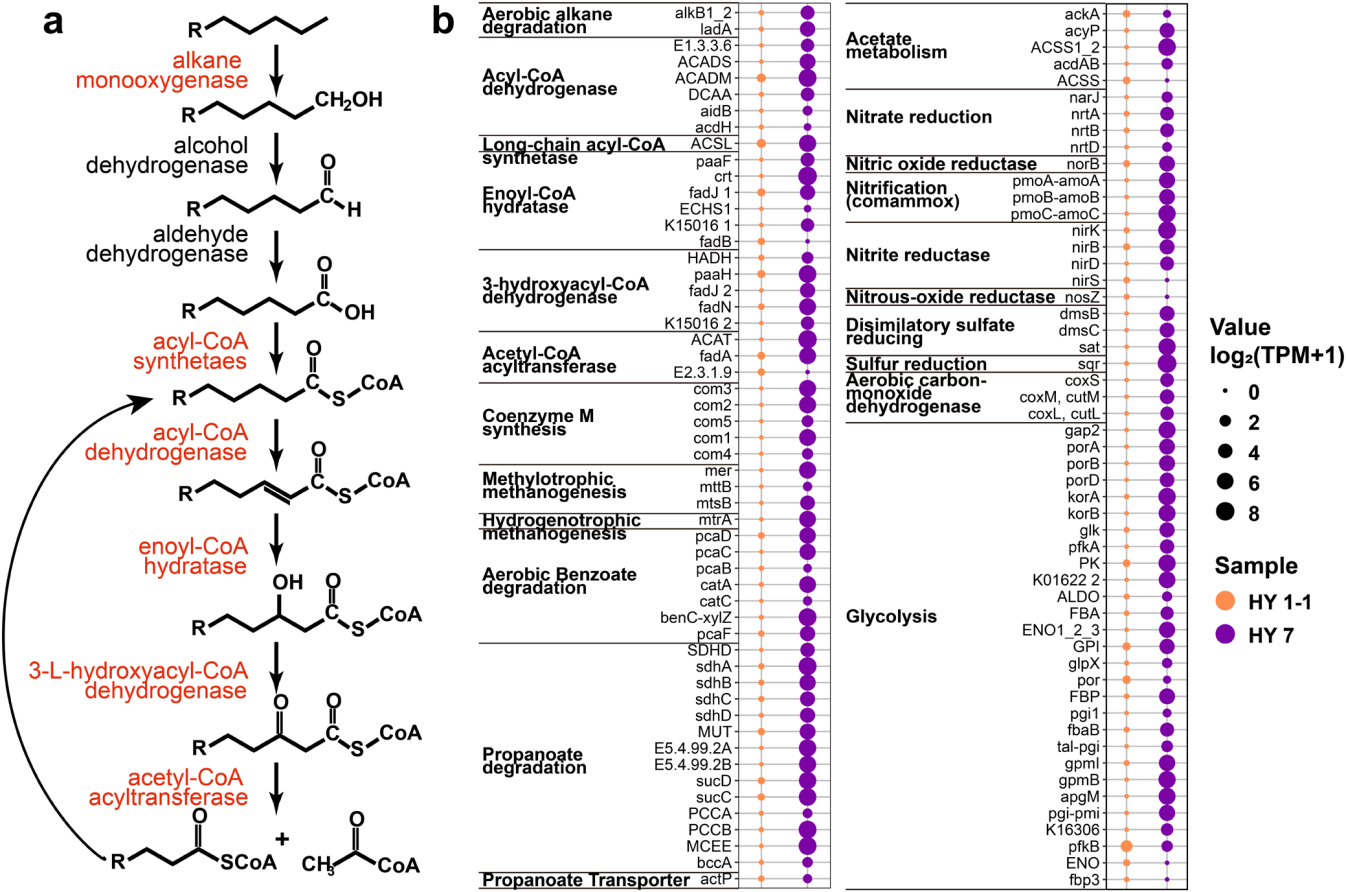
Aerobic degradation pathways of saturated hydrocarbons (a); Relative abundance (based on TPM) of functional genes predicted in KEGG pathway analysis within the microbial community in shale core (b). The TPM calculation involves three sequential steps: (1) Normalising read counts by gene length (in kilobases) to obtain reads per kilobase (RPK); (2) Summing all RPK values per sample and dividing by one million to derive the scaling factor; and (3) Dividing individual RPK values by this scaling factor to yield TPM.

The functional annotation results from the KEGG database indicated that the microbial community in both shale core samples possessed rich metabolic potential; however, most functional genes in HY7 were found in greater numbers than in HY1‐1 (Figure 5b, Table S8). The microbial community in HY7 was found to contain abundant glycolysis‐related functional genes. Additionally, genes related to aerobic hydrocarbon degradation and benzoate degradation were found in high abundance, suggesting that microorganisms may have utilised saturated hydrocarbons and aromatic hydrocarbons in crude oil as substrates for growth (Liu et al. 2018, 2019). However, most genes in the HY1‐1 sample were either unidentified or exhibited low TPM values. The *mtr*A gene, associated with hydrogenotrophic methanogenesis, as well as the *mtt*B, *mts*B and *mer* genes related to methylotrophic methanogenesis, were identified in the HY7 sample, along with the complete set of coenzyme M biosynthesis pathways, with all genes showing high TPM values except for *mttB*. In sulphur metabolism, functional genes related to dissimilatory sulphate reduction, including *sat*, *dms*C and *dms*B were detected in HY7. Meanwhile, the gene involved in sulphur reduction, *sqr*, was detected in both samples. Meanwhile, more abundant genes associated with nitrogen metabolism were found, including nitrate reduction (*nar*J, *nrt*A, *nrt*B and *nrt*D), nitrite reduction (*nir*K, *nir*B, *nir*D and *nir*S), nitrification (*pmo*A‐*amo*A, *pmo*B‐*amo*B and *pmo*C‐*amo*C), nitric oxide reduction (*nor*B), and nitrous oxide reductase (*nos*Z). The *nos*Z gene was detected exclusively in HY1‐1, whereas HY7 displayed higher completeness and relative abundance in other nitrogen metabolic functions.

## 4 Discussion

### 4.1 Shale Oil Reservoir Is a Habitat for Microbes

As a deep subsurface environment, shale oil reservoirs were extremely nutrient‐poor (Wu et al. 2015). While surfactants and other additives in fracturing fluids may function as microbial carbon sources and substantially modify community structures (Evans et al. 2019; Daly et al. 2016), the HY1‐1 and HY7 samples represent pre‐fracturing conditions, having been collected before any fluid injection occurred. Whether microorganisms can survive in such an oligotrophic environment is still an open question. Below, we will discuss the possibility from several aspects, including in situ water sources, physical space, carbon sources and energy sources.

Due to the small sample volume and difficulty in water extraction, the chemical composition of pore water was not analysed. It has been shown that all original shale reservoir formations contain primary water (Hu et al. 2019), primarily as capillary‐bound water in micropores smaller than 100 nm, while free water was found in macropores and fractures larger than 1000 nm. Clay minerals were more hydrophilic than organic matter and other inorganic minerals, and thus the majority of capillary‐bound water was tightly bound by them (Zhang, Lu, et al. 2024). In the sampled formation, clay minerals accounted for over 20% by mass (Figure 2c), which was consistent with Duan et al.'s (2024) finding that there were 30%–60% felsic minerals, 15%–55% carbonate minerals and 15%–45% clay minerals in the Funing Formation. Meanwhile, numerous macropores ranging from 1 to 10 μm were also present (Figure 2j). Consequently, it was believed that sufficient water was present in situ in the shale reservoir to support microbial survival. Although many studies had indicated the presence of water in shale, the lack of suitable markers to identify its source had prevented the determination of what additional substances this water might have provided for microbial growth.

The pore size in shale not only determined the distribution and transport rate of nutrients but also constrained the stochastic dispersal and chemotaxis of microorganisms (Xia et al. 2022). It was suggested that pore throats must be at least twice the diameter of microbial cells to allow the effective passage of bacteria (Updegraff 1983). Research by Yin et al. (2016) indicated that in shales with pore sizes predominantly ranging from 2 to 50 nm, physical impediments led to the cessation of microbial activity. Fu et al. (2024) have demonstrated that, aside from some intra‐granular dissolution pores which may be at the nanoscale, the majority of inter‐granular pore diameters were in the range of 1–2 μm. In our samples, over 70% of micropores had sizes exceeding 1 μm, with more than a quarter larger than 2 μm, and 2.4% and 3.8% of micropores were in the 5–10 μm range (Figure 2j), which met the minimum requirements for the growth of most microorganisms.

Another feature of shale was the presence of abundant organic compounds in the pores, with varying degrees of resistance to biodegradation, ordered from low to high as follows: *n*‐alkanes > *i‐alkanes > alkyl benzenes > alkyl naphthalenes > regular steranes > hopanes* > aromatic steranes (Elias et al. 2007). In this study, analysis of crude oil fractions indicated that saturated hydrocarbons accounted for more than 60% (Figure 3a). GC–MS results revealed that *n*‐alkanes were present in high concentrations, and the readily available *n*‐alkanes may provide a substantial carbon source for microbial growth.

Aside from carbon sources, nitrogen sources and inorganic salts were also crucial for microbial growth. Total nitrogen in shale was classified into organic and inorganic nitrogen. Organic nitrogen mainly existed in the forms of pyridine and quinoline (Zhu et al. 2021); meanwhile, some nitrogen entered the pore water as ammonium ions. When encountering K‐bearing minerals, inorganic nitrogen was predominantly fixed onto the minerals or adsorbed onto their surfaces, representing the primary form of inorganic nitrogen present (Meng et al. 2024). Since organic nitrogen compounds, including pyridine and quinoline, were predominantly distributed in resin and asphaltene fractions, their separation proved challenging, leading to their exclusion from identification. However, nitrogen‐containing organic compounds, including 3‐Methyl‐2‐benzothiazolinthion, Minaprine, Chloridazon and Vincamine, were detected in the GC–MS aromatic total ion chromatogram (Figure S3). In recent years, numerous studies have reported that microbial communities from shale oil reservoir samples exhibited active nitrogen metabolism functions (An et al. 2017; Morono et al. 2019). Besides organic compounds, minerals in shale reservoirs were also capable of providing essential elements and enzyme metal cofactors for microbial growth (Dong et al. 2022). Microorganisms were able to attach to mineral surfaces, promoting mineral dissolution through mineral‐microbe interactions. Alternatively, they lived in pore water as individuals or aggregates, utilising these elements by forming complexes with dissolved ions or through redox reactions (Ahmed and Holmström 2015), the latter also provided energy for the microorganisms (Dong et al. 2022). Minerals‐microbes' interactions were crucial to the global cycling of elements including Al, Si, Mg, Fe, P, S, C and N.

The distribution of live and dead bacteria on rock surfaces has been obtained from the microscope images. It can be observed from Figure 4a–d that a large amount of red and green fluorescence was detected on the surfaces of both samples. Additionally, numerous high‐intensity, relatively isolated ‘spot‐like’ signals with diameters of approximately 1 μm were present. This indicated the distribution of live and dead bacteria on the surfaces. However, a significant presence of aromatic hydrocarbons, resins and asphaltenes was detected in the crude oil extracted from the samples. The polycyclic aromatic hydrocarbons detected in both samples, including naphthalene, 2‐methylnaphthalene and 2,7‐dimethylnaphthalene, were also capable of fluorescence signal generation (Zhang, Yuan, et al. 2024) (Figure S4). Meanwhile, the adsorption of fluorescent dyes on the rock surface could not be excluded. Therefore, it was important to bear in mind that this interference might lead to an incorrect interpretation of the distribution, quantity and proportions of microorganisms on the sample surface.

Based on the above, the sampled shale reservoirs contained sufficient water, carbon sources, nutrients, physical space and energy for growth and reproduction. Meanwhile, numerous fluorescence signals, possibly associated with microorganisms, were detected on the core surface, indicating the shale oil reservoir was a potential microbial habitat.

### 4.2 Microorganisms in Shale Core Associated With Subsurface Carbon, Nitrogen and Sulphur Cycle

Many studies have shown that microorganisms can with stand extreme environmental stress through highly interdependent interactions (Pinheiro et al. 2023). While both HY1‐1 and HY7 exhibited considerable metabolic potential, their functional profiles diverged significantly, as evidenced by a Bray–Curtis dissimilarity of 0.89 (Table S7). Saturated and aromatic hydrocarbons were the most abundant carbon sources in shale reservoirs. In HY1‐1, *Marinobacter*, *Alcanivorax* and *Alkanindiges* were reported to contain the capability to degrade saturated hydrocarbons (Peña‐Montenegro et al. 2023; Dede et al. 2023; Woo et al. 2005), and in HY7, *Nocardioides* could degrade both saturated hydrocarbons and aromatic hydrocarbons (Ma et al. 2023). Many studies have reported the same hydrocarbon‐degrading microorganisms in shale oil reservoir environments (Evans et al. 2019; Daly et al. 2016; Ridley and Voordouw 2018). *Marinobacter* significantly upregulated the expression of *β*‐oxidation, chemotaxis genes and flagellar genes under oil spill perturbation, indicating that it was a good oil degrader (Peña‐Montenegro et al. 2023). A high abundance of *Alcanivorax* in the deep‐sea hydrothermal vents expressed a high abundance of genes involved in hydrocarbon degradation (Dede et al. 2023). *Nocardioides* have been shown to be well adapted to oligotrophic environments, where a variety of recalcitrant substances, including saturated hydrocarbons and aromatic hydrocarbons, were effectively degraded (Ma et al. 2023). In the absence of oxygen, hydrocarbon degradation typically requires alternative electron acceptors, such as SO_4_^2−^, NO_3_^−^ or Fe^3+^ (Zhang et al. 2019). In HY1‐1, the genera *Unclassified_Rhodocyclaceae*, *Halomonas* and *Unclassified_JG30‐KF‐CM45* may be involved in nitrogen metabolism (Shi et al. 2021; Wang and Shao 2021; Chen et al. 2024). *Sulfurimonas*, *Thiovirga*, *Desulfocapsa*, *Alishewanella* and *Unclassified_Desulfarculaceae* may be involved in sulphur metabolism (Molari et al. 2023; Ito et al. 2005; Rabus et al. 2015). Additionally, microorganisms with sulphur and nitrogen metabolism capabilities, such as *Unclassified_A4b* (Zhang et al. 2023), *Unclassified_JG30‐KF‐CM45* (Huang et al. 2022) and *Shewanella* (Li et al. 2023; Yu et al. 2021), were also found at relatively high abundances in HY7. An iron‐oxidising bacterium, *Unclassified_Caldilineaceae*, was identified in HY1‐1, which was an important component of iron‐oxidising microorganisms in marine environments, and its abundance was observed to decrease as oxygen concentrations increased (Frühe et al. 2021). HY1‐1 has an iron content of 6.57%, higher than the Earth's crust average of 5.6% (LePan and Venditti 2021). When oxygen availability was limited, the reduction of Fe^3+^, SO_4_^2−^ and NO_3_^−^ can couple with alkane degradation, thereby facilitating the efficient degradation of alkanes (Wartell et al. 2021). Meanwhile, corresponding functional genes related to hydrocarbon degradation, benzoate degradation, as well as sulphur and nitrogen metabolism were found in the HY7 sample, indicating that the microbial community possessed diverse metabolic function potential. Notably, methanogens were undetected in HY7 in the 16S rRNA gene survey, likely owing to their exceptionally low abundance. *Anoxybacillus* (Bremer et al. 2009) and *Thermobifida* (Deng et al. 2016) have both been reported to survive at temperatures of 60°C or even higher and possess the ability to degrade complex organic compounds. While pre‐collection sample storage may have modified microbial community structure and decreased thermophilic and anaerobic species abundance, the metabolic functions exhibited by resident microorganisms indicate adaptive survival traits for extreme native environments.

Glycolysis is a fundamental metabolic pathway responsible for the breakdown of glucose into two three‐carbon molecules of pyruvate. This process takes place in the cytosol of cells and is a metabolic pathway for ATP production shared by the vast majority of microorganisms (Kierans and Taylor 2024). The microbial community in HY7 possesses functional genes for the complete glycolytic metabolic pathway. Saturated hydrocarbons, which were abundant in shale oil, were identified as an important carbon source for in situ microbial growth. Saturated hydrocarbons were activated under the action of monooxygenases, and long‐chain fatty acids produced from primary alcohol oxidation enter the *β*‐oxidation pathway, ultimately generating water and carbon dioxide (Varjani 2017). In the absence of fracturing fluid components as alternative carbon sources, hydrocarbon degradation emerged as the primary heterotrophic carbon metabolism. The elevated TPM values demonstrate substantial potential for active hydrocarbon degradation under in situ conditions (Figure 5b). However, future studies based on meta‐transcriptomic profiling, enzymatic assays or stable isotope tracing are necessary to confirm active metabolic processes. The extremely low porosity of shale limits microbial activity; however, fermentation products from microorganisms in the shale layer can diffuse into the adjacent, more permeable sandstone layers. These substances support microbial growth and lead to the formation of larger microbial communities at the rock interface (Krumholz et al. 1997). At the same time, functional genes associated with denitrification (*nir*K, *nir*S, *nor*B) and dissimilatory nitrate reduction to ammonium (DNRA) (*nir*B, *nir*D) were annotated in HY7 and exhibited relatively high TPM values. Both denitrification and DNRA were more likely to occur in organic‐rich environments, which potentially facilitate the activation of fermentative microorganisms (Zhao et al. 2022). The enriched nitrite metabolism functions may suggest the accumulation of low‐concentration nitrite (Lim et al. 2018), which inhibited the expression of dissimilatory sulphite reductase (*Dsr*) in sulphate‐reducing bacteria (SRB) (An et al. 2017), potentially explaining the poor sulphate metabolism functions observed in the samples. Highly abundant ammonia‐oxidising functional genes (*pmo*A‐*amo*A, *pmo*B‐*amo*B and *pmo*C‐*amo*C) were identified in the HY7 sample. These genes facilitate the conversion of ammonium to hydroxylamine and significantly influence NO and N_2_O emissions through aerobic ammonia‐oxidising microorganisms (Soler‐Jofra et al. 2021). The genes *dmsB* and *dmsC* exhibit high abundance and participate in the dissimilatory sulphate reduction pathway, catalysing the conversion of dimethyl sulfoxide (DMSO) into dimethyl sulphide. Compared to conventional crude oil, shale oil contains higher levels of sulphur‐containing organic compounds, such as thiophenes, which can be converted into dimethyl sulfone—a precursor of DMSO (Mittal et al. 2016). The produced waters from wells HY1‐1 and HY7 exhibited sulphate concentrations of 4.8 and 1.1 mM, respectively, potentially serving as substrates for dissimilatory sulphate reduction. Abundant methyl compound metabolism functions, including both heterotrophic processes and methanogenesis, have been observed in both oceanic shale (Trembath‐Reichert et al. 2017) and produced water from shale oil reservoirs (An et al. 2019). While 16S rRNA amplicon sequencing did not detect methanogenic archaea, high‐depth metagenomic sequencing successfully identified several functional genes associated with methanogenesis. As discussed previously, this observed discrepancy may be attributed to either the relatively low abundance of methanogenic archaea or the limited sequencing depth achieved for the 16S rRNA amplicons. Meanwhile, the lack of replicate samples reduced the reliability of sequencing results; nevertheless, a more comprehensive analysis including more core samples is still required. Such occurrences have been documented between *Methanosarcinales* and *Crenarchaeota* (López‐García et al. 2004), as well as between *Methanosarcina* and cellulolytic bacteria (Rothman et al. 2014). Coenzyme M, which catalyses the final step of methanogenesis, can only be actively synthesised by a small number of microorganisms (Wu et al. 2022). The detection of multiple highly abundant methanogenic genes indicates that active hydrocarbon‐degrading methanogenesis likely occurs in shale oil reservoirs.

## 5 Conclusion

In conclusion, the results of this study demonstrate that the shale core of HY1‐1 and HY7 wells of Jiangsu Oilfield, which remain undisturbed by hydraulic fracturing or other oil extraction processes, met the necessary conditions for microbial growth, including water, carbon sources, nitrogen sources, trace elements and physical space. Shale oil reservoirs were identified as potential microbial habitats. The sample surfaces harboured numerous live and dead bacteria, with *Gammaproteobacteria* being identified as the most abundant class in both samples. Functional genes analysis revealed the presence of glycolysis, aerobic hydrocarbon degradation, as well as abundant nitrogen metabolism, and benzoate degradation functions were annotated. Overall, this study provided strong evidence for the presence of microorganisms in the pristine shale formations of oil reservoirs and identified the aerobic hydrocarbon degradation, glycolysis, methanogenesis, denitrification and DNRA functions of the microbial communities. Future studies should employ meta‐transcriptomic and stable isotope analyses to validate in situ microbial metabolic pathways within shale oil reservoirs.

## Author Contributions

**Bo‐Wen Wang:** writing – original draft, methodology, conceptualization, investigation, visualisation. **Yi‐Fan Liu:** methodology, conceptualization, supervision, writing – review and editing. **Le‐Gang Chen:** investigation, data curation. **Biao Wang:** investigation. **Zhi‐Hong Qian:** data curation, resources. **Fan Yang:** investigation, data curation. **Jia‐Cheng Cai:** investigation. **Lei Zhou:** conceptualization, supervision. **Shi‐Zhong Yang:** conceptualization, supervision. **Ji‐Dong Gu:** supervision. **Bo‐Zhong Mu:** conceptualization, project administration, supervision, funding acquisition, writing – review and editing.

## Conflicts of Interest

The authors declare no conflicts of interest.

## Supporting information


**Data S1:** Supporting information.

## Data Availability

The data that support the findings of this study are available at the NCBI Sequence Read Archive (https://www.ncbi.nlm.nih.gov/sra) under accession number PRJNA1224262.

## References

[emi470229-bib-0001] Ahmed, E. , and S. J. M. Holmström . 2015. “Microbe–Mineral Interactions: The Impact of Surface Attachment on Mineral Weathering and Element Selectivity by Microorganisms.” Chemical Geology 403: 13–23. 10.1016/j.chemgeo.2015.03.009.

[emi470229-bib-0002] Akob, D. M. , I. M. Cozzarelli , D. S. Dunlap , E. L. Rowan , and M. M. Lorah . 2015. “Organic and Inorganic Composition and Microbiology of Produced Waters From Pennsylvania Shale Gas Wells.” Applied Geochemistry 60: 116–125. 10.1016/j.apgeochem.2015.04.011.

[emi470229-bib-0003] Amundson, K. K. , M. A. Borton , R. A. Daly , et al. 2022. “Microbial Colonization and Persistence in Deep Fractured Shales Is Guided by Metabolic Exchanges and Viral Predation.” Microbiome 10: 5. 10.1186/s40168-022-01239-6. 35034639 PMC8762873

[emi470229-bib-0004] An, B. A. , Y. Shen , and G. Voordouw . 2017. “Control of Sulfide Production in High Salinity Bakken Shale Oil Reservoirs by Halophilic Bacteria Reducing Nitrate to Nitrite.” Frontiers in Microbiology 8: 1164. 10.3389/fmicb.2017.01164. 28680423 PMC5478722

[emi470229-bib-0005] An, B. A. , Y. Shen , J. Voordouw , and G. Voordouw . 2019. “Halophilic Methylotrophic Methanogens May Contribute to the High Ammonium Concentrations Found in Shale Oil and Shale Gas Reservoirs.” Frontiers in Energy Research 7: 23. 10.3389/fenrg.2019.00023.

[emi470229-bib-0006] Booker, A. E. , M. A. Borton , R. A. Daly , et al. 2017. “Sulfide Generation by Dominant Halanaerobium Microorganisms in Hydraulically Fractured Shales.” M Sphere 2, no. 2: e00257‐17. 10.1128/mspheredirect.00257-17. PMC5497025 28685163

[emi470229-bib-0007] Bremer, P. , B. Seale , S. Flint , and J. Palmer . 2009. “15–Biofilms in Dairy Processing.” In Biofilms in the Food and Beverage Industries, edited by P. M. Fratamico , B. A. Annous , and N. W. Gunther , 1st ed., 396–431. Woodhead Publishing.

[emi470229-bib-0008] Buchwalter, E. , J. A Swift , D. Sheets , et al. 2015. “Mapping of Microbial Habitats in Organic‐Rich Shale.” In Unconventional Resources Technology Conference, San Antonio. Society of Petroleum Engineers (SPE).

[emi470229-bib-0009] Chen, S. , Q.‐P. Zhang , J.‐S. Zhang , et al. 2024. “Enhanced Nitrogen Removal for Low C/N Wastewater via Preventing Futile Carbon Oxidation and Augmenting Anammox.” Water Research X 25: 100253. 10.1016/j.wroa.2024.100253. 39291147 PMC11405960

[emi470229-bib-0010] Cheng, Q. , M. Zhang , and H. Li . 2019. “Anomalous Distribution of Steranes in Deep Lacustrine Facies Low Maturity‐Maturity Source Rocks and Oil of Funing Formation in Subei Basin.” Journal of Petroleum Science and Engineering 181: 106190. 10.1016/j.petrol.2019.106190.

[emi470229-bib-0011] Chevalier, N. , N. Savoye , S. Dubois , et al. 2015. “Precise Indices Based on n‐Alkane Distribution for Quantifying Sources of Sedimentary Organic Matter in Coastal Systems.” Organic Geochemistry 88: 69–77. 10.1016/j.orggeochem.2015.07.006.

[emi470229-bib-0012] Cluff, M. A. , A. Hartsock , J. D. MacRae , K. Carter , and P. J. Mouser . 2014. “Temporal Changes in Microbial Ecology and Geochemistry in Produced Water From Hydraulically Fractured Marcellus Shale Gas Wells.” Environmental Science & Technology 48: 6508–6517. 10.1021/es501173p. 24803059

[emi470229-bib-0013] Colwell, F. S. , T. C. Onstott , M. E. Delwiche , et al. 1997. “Microorganisms From Deep, High Temperature Sandstones: Constraints on Microbial Colonization.” FEMS Microbiology Reviews 20: 425–435. 10.1016/S0168-6445(97)00024-7.

[emi470229-bib-0014] Daly, R. A. , M. A. Borton , M. J. Wilkins , et al. 2016. “Microbial Metabolisms in a 2.5‐km‐Deep Ecosystem Created by Hydraulic Fracturing in Shales.” Nature Microbiology 1: 16146. 10.1038/nmicrobiol.2016.146. 27595198

[emi470229-bib-0015] Dede, B. , T. Priest , W. Bach , M. Walter , R. Amann , and A. Meyerdierks . 2023. “High Abundance of Hydrocarbon‐Degrading Alcanivorax in Plumes of Hydrothermally Active Volcanoes in the South Pacific Ocean.” ISME Journal 17: 600–610. 10.1038/s41396-023-01366-4. 36721059 PMC10030979

[emi470229-bib-0016] Deng, Y. , J. Lin , Y. Mao , and X. Zhang . 2016. “Systematic Analysis of an Evolved *Thermobifida* fusca muC Producing Malic Acid on Organic and Inorganic Nitrogen Sources.” Scientific Reports 6: 30025. 10.1038/srep30025. 27424527 PMC4948018

[emi470229-bib-0017] Didyk, B. M. , B. R. T. Simoneit , S. C. Brassell , and G. Eglinton . 1978. “Organic Geochemical Indicators of Palaeoenvironmental Conditions of Sedimentation.” Nature 272: 216–222. 10.1038/272216a0.

[emi470229-bib-0018] Dong, H. , L. Huang , L. Zhao , et al. 2022. “A Critical Review of Mineral‐Microbe Interaction and Co‐Evolution: Mechanisms and Applications.” National Science Review 9: nwac128. 10.1093/nsr/nwac128. 36196117 PMC9522408

[emi470229-bib-0019] Duan, H. L. , Y. X. Sun , and B. L. Yang . 2024. “Main Controlling Factors of Shale Oil Enrichment in Second Member of Paleogene Funing Formation in Gaoyou Sag of Subei Basin.” Petroleum Geology & Experiment 46: 441–450. 10.11781/sysydz202403441.

[emi470229-bib-0020] Elias, R. , A. Vieth , A. Riva , B. Horsfield , and H. Wilkes . 2007. “Improved Assessment of Biodegradation Extent and Prediction of Petroleum Quality.” Organic Geochemistry 38: 2111–2130. 10.1016/j.orggeochem.2007.07.004.

[emi470229-bib-0021] Evans, M. V. , G. Getzinger , J. L. Luek , et al. 2019. “In Situ Transformation of Ethoxylate and Glycol Surfactants by Shale‐Colonizing Microorganisms During Hydraulic Fracturing.” ISME Journal 13: 2690–2700. 10.1038/s41396-019-0466-0. 31243331 PMC6794257

[emi470229-bib-0022] Fredrickson, J. K. , J. P. McKinley , B. N. Bjornstad , et al. 1997. “Pore‐Size Constraints on the Activity and Survival of Subsurface Bacteria in a Late Cretaceous Shale‐Sandstone Sequence, Northwestern New Mexico.” Geomicrobiology Journal 14: 183–202. 10.1080/01490459709378043.

[emi470229-bib-0023] Frühe, L. , V. Dully , D. Forster , et al. 2021. “Global Trends of Benthic Bacterial Diversity and Community Composition Along Organic Enrichment Gradients of Salmon Farms.” Frontiers in Microbiology 12: 637811. 10.3389/fmicb.2021.637811. 33995296 PMC8116884

[emi470229-bib-0024] Fu, Q. , H. L. Duan , and S. Liu . 2024. “Study on Pore Throat Structure Characteristics of Shale Reservoirs in the Second Member of Funing Formation in the Huazhuang Area of Gaoyou Sag.” Complex Hydrocarbon Reservoirs 17: 131–138. 10.16181/j.cnki.fzyqc.2024.02.001.

[emi470229-bib-0025] Hernandez‐Becerra, N. , L. Cliffe , W. Xiu , C. Boothman , J. R. Lloyd , and S. L. Nixon . 2023. “New Microbiological Insights From the Bowland Shale Highlight Heterogeneity of the Hydraulically Fractured Shale Microbiome.” Environmental Microbiology 18: 14. 10.1186/s40793-023-00465-1. PMC9972762 36855215

[emi470229-bib-0026] Hoehler, T. M. , and B. B. Jørgensen . 2013. “Microbial Life Under Extreme Energy Limitation.” Nature Reviews. Microbiology 11: 83–94. 10.1038/nrmicro2939. 23321532

[emi470229-bib-0027] Hu, Z. , X. Duan , Y. He , et al. 2019. “Influence of Reservoir Primary Water on Shale Gas Occurrence and Flow Capacity.” Natural Gas Industry B 6: 71–78. 10.1016/j.ngib.2019.01.010.

[emi470229-bib-0028] Huang, X. , Y. Xing , H. Wang , Z. Dai , and T. Chen . 2022. “Nitrogen Advanced Treatment of Urban Sewage by Denitrification Deep‐Bed Filter: Removal Performance and Metabolic Pathway.” Frontiers in Microbiology 12: 811697. 10.3389/fmicb.2021.811697. 35154036 PMC8825488

[emi470229-bib-0029] Hyatt, D. , G.‐L. Chen , P. F. LoCascio , M. L. Land , F. W. Larimer , and L. J. Hauser . 2010. “Prodigal: Prokaryotic Gene Recognition and Translation Initiation Site Identification.” BMC Bioinformatics 11: 119. 10.1186/1471-2105-11-119. 20211023 PMC2848648

[emi470229-bib-0030] Ito, T. , K. Sugita , I. Yumoto , Y. Nodasaka , and S. Okabe . 2005. “ Thiovirga sulfuroxydans gen. nov., sp. nov., a Chemolithoautotrophic Sulfur‐Oxidizing Bacterium Isolated From a Microaerobic Waste‐Water Biofilm.” International Journal of Systematic and Evolutionary Microbiology 55: 1059–1064. 10.1099/ijs.0.63467-0. 15879233

[emi470229-bib-0031] Jiang, Z. , W. Zhang , C. Liang , Y. Wang , H. Liu , and X. Chen . 2016. “Basic Characteristics and Evaluation of Shale Oil Reservoirs.” Petroleum Research 1: 149–163. 10.1016/S2096-2495(17)30039-X.

[emi470229-bib-0032] Kierans, S. J. , and C. T. Taylor . 2024. “Glycolysis: A Multifaceted Metabolic Pathway and Signaling Hub.” Journal of Biological Chemistry 300: 107906. 10.1016/j.jbc.2024.107906. 39442619 PMC11605472

[emi470229-bib-0033] Krumholz, L. R. , J. P. McKinley , G. A. Ulrich , and J. M. Suflita . 1997. “Confined Subsurface Microbial Communities in Cretaceous Rock.” Nature 386: 64–66. 10.1038/386064a0.

[emi470229-bib-0034] LePan, N. , and B. Venditti . 2021. Visualizing the Abundance of Elements in the Earth's Crust. World Economin Forum.

[emi470229-bib-0035] Li, H. , X. Zhang , Y. Zhang , et al. 2023. “The Iron Cycling Mediated by a Single Strain *Shewanella oneidensis* MR‐1 and Its Implication for Nitrogen Removal.” Chemical Engineering Journal 471: 144727. 10.1016/j.cej.2023.144727.

[emi470229-bib-0036] Lim, N. Y. N. , Å. Frostegård , and L. R. Bakken . 2018. “Nitrite Kinetics During Anoxia: The Role of Abiotic Reactions Versus Microbial Reduction.” Soil Biology and Biochemistry 119: 203–209. 10.1016/j.soilbio.2018.01.006.

[emi470229-bib-0037] Liu, Y.‐F. , D. D. Galzerani , S. M. Mbadinga , et al. 2018. “Metabolic Capability and In Situ Activity of Microorganisms in an Oil Reservoir.” Microbiome 6: 5. 10.1186/s40168-017-0392-1. 29304850 PMC5756336

[emi470229-bib-0038] Liu, Y.‐F. , Z.‐Z. Qi , L.‐B. Shou , et al. 2019. “Anaerobic Hydrocarbon Degradation in Candidate Phylum ‘Atribacteria’ (JS1) Inferred From Genomics.” ISME Journal 13: 2377–2390. 10.1038/s41396-019-0448-2. 31171858 PMC6776118

[emi470229-bib-0039] López‐García, P. , C. Brochier , D. Moreira , and F. Rodríguez‐Valera . 2004. “Comparative Analysis of a Genome Fragment of an Uncultivated Mesopelagic Crenarchaeote Reveals Multiple Horizontal Gene Transfers.” Environmental Microbiology 6: 19–34. 10.1046/j.1462-2920.2003.00533.x. 14686938

[emi470229-bib-0040] Luo, Y. , and Y. J. Xing . 2023. “Study on Source Rock Characteristics and Oil Bearing Properties of the Second Section of Funing Formation in Huazhuang Area of Gaoyou Sag.” In International Field Exploration and Development Conference, Wu Han, China. Springer Nature.

[emi470229-bib-0041] Ma, Y. , J. Wang , Y. Liu , et al. 2023. “Nocardioides: “Specialists” for Hard‐to‐Degrade Pollutants in the Environment.” Molecules 28: 7433. 10.3390/molecules28217433. 37959852 PMC10649934

[emi470229-bib-0042] Meng, G. , H. Gai , X. Yang , et al. 2024. “Occurrence and Maturation Transformation of Organic and Inorganic Nitrogen in the Lower Cambrian Shelf–Slope Facies Shale: Implications for Overmature N2‐Rich Shale Reservoirs in Southern China.” Marine and Petroleum Geology 168: 107011. 10.1016/j.marpetgeo.2024.107011.

[emi470229-bib-0043] Mittal, S. , M. Garg , and P. Sande . 2016. “Characterization and Comparison of Shale Oil and Crude Oil.” In 6th World Petro Coal Conference, New Delhi, India. Energy and Environment Foundation (India).

[emi470229-bib-0044] Molari, M. , C. Hassenrueck , R. Laso‐Pérez , et al. 2023. “A Hydrogenotrophic Sulfurimonas Is Globally Abundant in Deep‐Sea Oxygen‐Saturated Hydrothermal Plumes.” Nature Microbiology 8: 651–665. 10.1038/s41564-023-01342-w. PMC10066037 36894632

[emi470229-bib-0045] Morono, Y. , J. R. Wishart , M. Ito , et al. 2019. “Microbial Metabolism and Community Dynamics in Hydraulic Fracturing Fluids Recovered From Deep Hydrocarbon‐Rich Shale.” Frontiers in Microbiology 10: 376. 10.3389/fmicb.2019.00376. 30915042 PMC6422894

[emi470229-bib-0046] Mouser, P. J. , M. Borton , T. H. Darrah , A. Hartsock , and K. C. Wrighton . 2016. “Hydraulic Fracturing Offers View of Microbial Life in the Deep Terrestrial Subsurface.” FEMS Microbiology Ecology 92: fiw166. 10.1093/femsec/fiw166. 27507739

[emi470229-bib-0047] Onstott, T. , T. Phelps , F. Colwell , et al. 1998. “Observations Pertaining to the Origin and Ecology of Microorganisms Recovered From the Deep Subsurface of Taylorsville Basin, Virginia.” Geomicrobiology Journal 15: 353–385. 10.1080/01490459809378088.

[emi470229-bib-0048] Peña‐Montenegro, T. D. , S. Kleindienst , A. E. Allen , et al. 2023. “Species‐Specific Responses of Marine Bacteria to Environmental Perturbation.” ISME Communications 3: 99. 10.1038/s43705-023-00310-z. 37736763 PMC10516948

[emi470229-bib-0049] Peters, K. E. , and J. M. Moldowan . 1992. The Biomarker Guide: Interpreting Molecular Fossils in Petroleum and Ancient Sediments. Prentice Hall.

[emi470229-bib-0050] Pinheiro, Y. , F. F. da Mota , R. S. Peixoto , et al. 2023. “A Thermophilic Chemolithoautotrophic Bacterial Consortium Suggests a Mutual Relationship Between Bacteria in Extreme Oligotrophic Environments.” Communications Biology 6: 230. 10.1038/s42003-023-04617-4. 36859706 PMC9977764

[emi470229-bib-0051] Rabus, R. , S. S. Venceslau , L. Wöhlbrand , G. Voordouw , J. D. Wall , and I. A. C. Pereira . 2015. “Chapter Two–A Post‐Genomic View of the Ecophysiology, Catabolism and Biotechnological Relevance of Sulphate‐Reducing Prokaryotes.” Advances in Microbial Physiology 66: 55–321. 10.1016/bs.ampbs.2015.05.002. 26210106

[emi470229-bib-0052] Ridley, C. M. , and G. Voordouw . 2018. “Aerobic Microbial Taxa Dominate Deep Subsurface Cores From the Alberta Oil Sands.” FEMS Microbiology Ecology 94: fiy073. 10.1093/femsec/fiy073. 29688331

[emi470229-bib-0053] Rothman, D. H. , G. P. Fournier , K. L. French , et al. 2014. “Methanogenic Burst in the End‐Permian Carbon Cycle.” PNAS 111: 5462–5467. 10.1073/pnas.1318106111. 24706773 PMC3992638

[emi470229-bib-0054] Schlegel, M. E. , J. C. McIntosh , S. T. Petsch , W. H. Orem , E. J. P. Jones , and A. M. Martini . 2013. “Extent and Limits of Biodegradation by In Situ Methanogenic Consortia in Shale and Formation Fluids.” Applied Geochemistry 28: 172–184. 10.1016/j.apgeochem.2012.10.008.

[emi470229-bib-0055] Sharma, S. , T. R. Carr , P. J. Mouser , et al. 2017. “Biogeochemical Characterization of Core, Fluids, and Gas at MSEEL Site.” In Unconventional Resources Technology Conference. Society of Petroleum Engineers (SPE).

[emi470229-bib-0056] Shi, L.‐D. , P.‐L. Lv , S. J. McIlroy , et al. 2021. “Methane‐Dependent Selenate Reduction by a Bacterial Consortium.” ISME Journal 15: 3683–3692. 10.1038/s41396-021-01044-3. 34183781 PMC8630058

[emi470229-bib-0057] Shu, W.‐S. , and L.‐N. Huang . 2022. “Microbial Diversity in Extreme Environments.” Nature Reviews. Microbiology 20: 219–235. 10.1038/s41579-021-00648-y. 34754082

[emi470229-bib-0058] Soler‐Jofra, A. , J. Pérez , and M. C. M. van Loosdrecht . 2021. “Hydroxylamine and the Nitrogen Cycle: A Review.” Water Research 190: 116723. 10.1016/j.watres.2020.116723. 33352529

[emi470229-bib-0059] Stolz, J. F. , C. Ziegler , and W. M. Griffin . 2022. “Global Unconventional Oil and Gas Reserves and Their Development.” In Environmental Impacts From the Development of Unconventional Oil and Gas Reserves, edited by J. Stolz , D. Bain , and M. Griffin , 1st ed., 3–18. Cambridge University Press.

[emi470229-bib-0060] Tinker, K. , J. Gardiner , D. Lipus , P. Sarkar , M. Stuckman , and D. Gulliver . 2020. “Geochemistry and Microbiology Predict Environmental Niches With Conditions Favoring Potential Microbial Activity in the Bakken Shale.” Frontiers in Microbiology 11: 1781. 10.3389/fmicb.2020.01781. 32849400 PMC7406717

[emi470229-bib-0061] Trembath‐Reichert, E. , Y. Morono , A. Ijiri , et al. 2017. “Methyl‐Compound Use and Slow Growth Characterize Microbial Life in 2‐km‐Deep Subseafloor Coal and Shale Beds.” PNAS 114: E9206–E9215. 10.1073/pnas.1707525114. 29078310 PMC5676895

[emi470229-bib-0062] Trexler, R. V. 2017. Lipid Analysis and Microbial Community Characterization of Subsurface Shale. Dissertation, The Ohio State University.

[emi470229-bib-0063] Tsesmetzis, N. , E. B. Alsop , A. Vigneron , F. Marcelis , I. M. Head , and B. P. Lomans . 2018. “Microbial Community Analysis of Three Hydrocarbon Reservoir Cores Provides Valuable Insights for the Assessment of Reservoir Souring Potential.” International Biodeterioration and Biodegradation 126: 177–188. 10.1016/j.ibiod.2016.09.002.

[emi470229-bib-0064] Tucker, Y. T. , J. Kotcon , and T. Mroz . 2015. “Methanogenic Archaea in Marcellus Shale: A Possible Mechanism for Enhanced Gas Recovery in Unconventional Shale Resources.” Environmental Science & Technology 49: 7048–7055. 10.1021/acs.est.5b00765. 25924080

[emi470229-bib-0065] Tucker, Y. T. , and T. Mroz . 2018. “Microbes in Marcellus Shale: Extremophiles Living More Than Two Kilometers Inside the Earth?” Fuel 234: 1205–1211. 10.1016/j.fuel.2018.06.125.

[emi470229-bib-0066] Updegraff, D. M. 1983. “Plugging and Penetration of Petroleum Reservoir Rock by Microorganisms.” In International Conference on Microbial Enhancement of Oil Recovery, Norman, United States. United States Department of Energy.

[emi470229-bib-0067] Varjani, S. J. 2017. “Microbial Degradation of Petroleum Hydrocarbons.” Bioresource Technology 223: 277–286. 10.1016/j.biortech.2016.10.037. 27789112

[emi470229-bib-0068] Wang, L. , and Z. Shao . 2021. “Aerobic Denitrification and Heterotrophic Sulfur Oxidation in the Genus Halomonas Revealed by Six Novel Species Characterizations and Genome‐Based Analysis.” Frontiers in Microbiology 12: 652766. 10.3389/fmicb.2021.652766. 33815342 PMC8014003

[emi470229-bib-0069] Wang, M. , Z. Guo , C. Jiao , et al. 2019. “Exploration Progress and Geochemical Features of Lacustrine Shale Oils in China.” Journal of Petroleum Science and Engineering 178: 975–986. 10.1016/j.petrol.2019.04.029.

[emi470229-bib-0070] Wartell, B. , M. Boufadel , and L. Rodriguez‐Freire . 2021. “An Effort to Understand and Improve the Anaerobic Biodegradation of Petroleum Hydrocarbons: A Literature Review.” International Biodeterioration and Biodegradation 157: 105156. 10.1016/j.ibiod.2020.105156.

[emi470229-bib-0071] Wentzel, A. , A. Lewin , F. J. Cervantes , S. Valla , and H. K. Kotlar . 2013. “Deep Subsurface Oil Reservoirs as Poly‐Extreme Habitats for Microbial Life. A Current Review.” In Polyextremophiles: Life Under Multiple Forms of Stress, edited by J. Seckbach , A. Oren , and H. Stan‐Lotter , 1st ed., 439–466. Springer Netherlands.

[emi470229-bib-0072] Wen‐Yen, H. , and W. G. Meinschein . 1976. “Sterols as Source Indicators of Organic Materials in Sediments.” Geochimica et Cosmochimica Acta 40: 323–330. 10.1016/0016-7037(76)90210-6.

[emi470229-bib-0073] Woo, P. C. , H. Tse , S. K. Lau , et al. 2005. “Alkanindiges *Hongkongensis* sp. nov. a Novel Alkanindiges Species Isolated From a Patient With Parotid Abscess.” Systematic and Applied Microbiology 28: 316–322. 10.1016/j.syapm.2005.01.003. 15997704

[emi470229-bib-0074] Wu, H.‐H. , M. D. Pun , C. E. Wise , et al. 2022. “The Pathway for Coenzyme M Biosynthesis in Bacteria.” PNAS 119: e2207190119. 10.1073/pnas.2207190119. 36037354 PMC9457059

[emi470229-bib-0075] Wu, X. , K. Holmfeldt , V. Hubalek , et al. 2015. “Microbial Metagenomes From Three Aquifers in the Fennoscandian Shield Terrestrial Deep Biosphere Reveal Metabolic Partitioning Among Populations.” ISME Journal 10: 1192–1203. 10.1038/ismej.2015.185. 26484735 PMC5029217

[emi470229-bib-0076] Xia, Q. , N. Zheng , J. L. Heitman , and W. Shi . 2022. “Soil Pore Size Distribution Shaped Not Only Compositions but Also Networks of the Soil Microbial Community.” Applied Soil Ecology 170: 104273. 10.1016/j.apsoil.2021.104273.

[emi470229-bib-0077] Xiang, S. , C. Xiang , Z. Xinwen , J. Yanyu , and L. Xi . 2015. “Prospects and Challenges of Continental Shale Oil Development in China.” Petroleum Geology & Experiment 37: 267–271. 10.11781/sysydz201503267.

[emi470229-bib-0078] Yin, M. , H. Huang , and J. Ma . 2016. “Pore Size Constrains on Hydrocarbon Biodegradation in Shales From the Second White Speckled Shale Formation of the Western Canada Sedimentary Basin.” Fuel 185: 639–648. 10.1016/j.fuel.2016.08.034.

[emi470229-bib-0079] Yu, Q. , W. Sun , and H. Gao . 2021. “Thiosulfate Oxidation in Sulfur‐Reducing Shewanella Oneidensis and Its Unexpected Influences on the Cytochrome c Content.” Environmental Microbiology 23: 7056–7072. 10.1111/1462-2920.15807. 34664382

[emi470229-bib-0080] Zhang, K. , Z. Hu , F. Zeng , et al. 2019. “Biodegradation of Petroleum Hydrocarbons and Changes in Microbial Community Structure in Sediment Under Nitrate‐, Ferric‐, Sulfate‐Reducing and Methanogenic Conditions.” Journal of Environmental Management 249: 109425. 10.1016/j.jenvman.2019.109425. 31446121

[emi470229-bib-0081] Zhang, P.‐F. , S.‐F. Lu , J.‐J. Wang , et al. 2024. “Microscopic Occurrence and Distribution of Oil and Water In Situ Shale: Evidence From Nuclear Magnetic Resonance.” Petroleum Science 21: 3675–3691. 10.1016/j.petsci.2024.04.007.

[emi470229-bib-0082] Zhang, S. , Y. Yuan , Z. Wang , and J. Li . 2024. “The Application of Laser‐Induced Fluorescence in Oil Spill Detection.” Environmental Science and Pollution Research 31: 23462–23481. 10.1007/s11356-024-32807-y. 38466385

[emi470229-bib-0083] Zhang, Y. , Y. Qiao , and Z. Fu . 2023. “Shifts of Bacterial Community and Predictive Functional Profiling of Denitrifying Phosphorus Removal—Partial Nitrification—Anammox Three‐Stage Nitrogen and Phosphorus Removal Before and After Coupling for Treating Simulated Wastewater With Low C/N.” Chemical Engineering Journal 451: 138601. 10.1016/j.cej.2022.138601.

[emi470229-bib-0084] Zhao, Y. , Q. Li , Q. Cui , and S.‐Q. Ni . 2022. “Nitrogen Recovery Through Fermentative Dissimilatory Nitrate Reduction to Ammonium (DNRA): Carbon Source Comparison and Metabolic Pathway.” Chemical Engineering Journal 441: 135938. 10.1016/j.cej.2022.135938.

[emi470229-bib-0085] Zhu, G. , F. Xing , J. Tao , et al. 2021. “Synergy of Strains That Accelerate Biodegradation of Pyridine and Quinoline.” Journal of Environmental Management 285: 112119. 10.1016/j.jenvman.2021.112119. 33581454

[emi470229-bib-0086] Zou, C. , Z. Yang , J. Cui , et al. 2013. “Formation Mechanism, Geological Characteristics and Development Strategy of Nonmarine Shale Oil in China.” Petroleum Exploration and Development 40: 15–27. 10.1016/S1876-3804(13)60002-6.

