# Supplementary material for: Microbial Community Composition and Function in Jiangsu Oil Reservoir Cores, China
Source: Environ Microbiol Rep. 2025 Nov 26;17(6):e70229. doi: 10.1111/1758-2229.70229 (PMC12657123; doi:10.1111/1758-2229.70229)
Supplement: Supplementary file 1 — Data S1: Supporting information. [file EMI4-17-e70229-s001.docx]

***Supplementary Information for***

**Microbial Community Composition and Function in Jiangsu Oil Reservoir Cores, China**

Bo-Wen Wang^a, b^, Yi-Fan Liu^a, b, *^, Le-Gang Chen^a, b^, Biao Wang^c^, Zhi-Hong Qian^c^, Fan Yang^c^, Jia-Cheng Cai^d^, Lei Zhou^a, b^, Shi-Zhong Yang^a, b^, Ji-Dong Gu^e^ and Bo-Zhong Mu^a, b, *^

^a^ State Key Laboratory of Bioreactor Engineering and School of Chemistry and Molecular Engineering, East China University of Science and Technology, 130 Meilong Road, Shanghai 200237, P.R. China

^b^ Engineering Research Center of MEOR, East China University of Science and Technology, 130 Meilong Road, Shanghai 200237, P. R. China

^c^ Research Institute of Petroleum Engineering, Jiangsu Oilfield, Sinopec, Jiangsu 225007, P. R. China

^d^ College of Chemistry and Molecular Engineering, East China University of Science and Technology, 130 Meilong Road, Shanghai 200237, P.R. China

^e^ Environmental Science and Engineering Group, Guangdong Technion Israel Institute of Technology, 241 Daxue Road, Shantou, Guangdong 515063, P.R. China

Bo-Wen Wang, [bwwang@mail.ecust.edu.cn](mailto:bwwang@mail.ecust.edu.cn), <https://orcid.org/0009-0000-5398-4646>

Le-Gang Chen, <legangchen@mail.ecust.edu.cn>, <https://orcid.org/0009-0006-0021-0819>

Biao Wang, [wangbiao.jsyt@sinopec.com](mailto:wangbiao.jsyt@sinopec.com), <https://orcid.org/0009-0004-7560-8958>

Zhi-Hong Qian, [qzhgoon@163.com](mailto:qzhgoon@163.com), <https://orcid.org/0009-0008-6333-1057>

Fan Yang, [yangfan.jsyt@sinopec.com](mailto:yangfan.jsyt@sinopec.com), <https://orcid.org/0009-0003-1518-9280>

Jia-Cheng Cai, [1969175020@qq.com](mailto:1969175020@qq.com), <https://orcid.org/0009-0000-0950-6575>

Lei Zhou, [leizhou@ecust.edu.cn](mailto:leizhou@ecust.edu.cn), <https://orcid.org/0000-0002-9301-7770>

Shi-Zhong Yang, [meor@ecust.edu.cn](mailto:meor@ecust.edu.cn), <https://orcid.org/0000-0001-9979-4469>

Ji-Dong Gu, [jidong.gu@gtiit.edu.cn](mailto:jidong.gu@gtiit.edu.cn), <https://orcid.org/0000-0002-7082-9784>

^*^ Address correspondence to Bo-Zhong Mu, [bzmu@ecust.edu.cn](mailto:bzmu@ecust.edu.cn), <https://orcid.org/0000-0002-9564-4970>

Or Yi-Fan Liu, [liuyifan@ecust.edu.cn](mailto:liuyifan@ecust.edu.cn), <https://orcid.org/0000-0001-5048-198X>

**Materials and methods**

**The trimmed inner rock (TIR) powder preparation.** In the clean benches, the TIR was placed in a beaker and submerged in a sterile PBS buffer. The beaker was sealed with parafilm and incubated on a shaking incubator at 37 °C for 30 minutes to remove rock dust on the surface. After incubation, the samples were air-dried in the clean benches and exposed to UV light for 30 minutes. The cleaned rock samples were then pulverized using a mineral grinder at 28000 r/min which had been sterilized with 75% alcohol in advance. The TIR powder was transferred to 40 mL sterile centrifuge tubes and stored at 4 °C until DNA extraction.

**The trimmed inner rock pieces preparation.** The TIR was sliced into thin pieces using sterile blades in clean benches and placed into sterile centrifuge tubes.

**The trimmed outer rock (TOR) powder preparation.** The TOR was washed with deionized water to remove surface dust, air-dried, and then directly pulverized to obtain TOR powder.

**Table S1. Relative abundance (*wt*.%) of elements in shale cores**

Element HY1-1 HY7 Element HY1-1 HY7

Al 15.74 12.1 Mn 0.0631 0.0691

As 0.0052 0.0038 Na 3.45 4.58

Ba 0 0.0457 Ni 0.0034 0.0023

Ca 4.27 9.49 Rb 0.021 0.0101

Cl 0 0.0001 S 3.2 1.77

Cr 0.0144 0.0088 Si 58.85 62.86

Cu 0.0033 0.0015 Sr 0.0569 0.1184

Fe 6.57 4.2 Ti 0.7421 0.5611

Ga 0.0063 0.0042 V 0.0194 0

K 3.75 2.31 Zn 0.014 0.0097

Mg 3.2 1.81 Zr 0.0241 0.0408

**Table S2. Extraction of organic matter from shale core and separation of crude oil family composition**

Sample Shacle core/g Family composition/g Content, wt.%

HY1-1 25.0047 0.0242 0.0963

HY7 37.6483 0.0276 0.0733

**Table S3.** **Mass composition of crude oil family composition**

Sample HY1-1 HY7

Family composition/g 0.0242 0.0276

Saturated hydrocarbon Weight/g 0.0145 0.0205

Content, wt.% 59.92 74.28

Aromatic hydrocarbon Weight/g 0.0018 0.0016

Content, wt.% 7.44 5.80

Resin Weight/g 0.0051 0.0029

Content, wt.% 21.07 10.51

Asphaltene Weight/g 0.0028 0.0026

Content, wt.% 11.57 9.42

**Table S4. The peak areas of the conventional steranes C_27_, C_28_, and C_29_**

Area

Carbon number Name HY1-1 HY7

C27 20R-ααα-cholestane 29029.765 52146.949

C28 20R-24-methyl-ααα-cholestane 16156.016 32158.851

C29 20R-24-ethyl-ααα-cholestane 25735.632 51106.23

**Table S5. Relative abundance (%) of all bacterial genera in the HY1-1.**

Genus HY1-1 Genus HY1-1

Pseudomonas 35.29 Unclassified_Coleofasciculaceae 0.09

Unclassified_Enterobacteriaceae 6.81 Unclassified_Saccharimonadales 0.09

Acinetobacter 3.51 Duganella 0.09

Unclassified_Planococcaceae 2.10 Mycobacterium 0.08

Comamonas 1.97 Brachybacterium 0.08

Stenotrophomonas 1.86 Unclassified_Burkholderiales 0.08

Pedobacter 1.82 Cellvibrio 0.08

Hydrogenophaga 1.45 Actinomycetospora 0.08

Unclassified_Saccharospirillaceae 1.32 Pontibacter 0.08

Bacillus 1.30 Rheinheimera 0.08

Sulfurimonas 1.21 Rhodanobacter 0.08

Unclassified_Enterobacterales 1.16 Unclassified_GIF9 0.08

Alteromonas 1.09 Unclassified_Solimonadaceae 0.08

Brevundimonas 1.02 Unclassified_Chitinophagaceae 0.08

Sphingomonas 0.99 Chitinivorax 0.08

Marinobacter 0.92 Clostridium sensu stricto 8 0.08

Exiguobacterium 0.88 Unclassified_env.OPS 17 0.08

Unclassified_Comamonadaceae 0.88 Xanthobacter 0.08

Dietzia 0.87 Pleurocapsa PCC-7319 0.07

Unclassified_Rhodocyclaceae 0.77 Desemzia 0.07

Methylobacterium-Methylorubrum 0.75 Elizabethkingia 0.07

Ornithinimicrobium 0.72 Ensifer 0.07

Unclassified_Alcaligenaceae 0.70 Escherichia-Shigella 0.07

Rhodococcus 0.68 Nocardia 0.07

Corynebacterium 0.66 Solitalea 0.07

Novosphingobium 0.61 YC-ZSS-LKJ147 0.07

Nesterenkonia 0.61 Unclassified_Gammaproteobacteria 0.06

Alcanivorax 0.60 Blastococcus 0.06

Planococcus 0.57 Blautia 0.06

Halomonas 0.57 Conexibacter 0.06

Delftia 0.55 Modestobacter 0.06

Unclassified_Sphingomonadaceae 0.54 Neomicrococcus 0.06

Thiovirga 0.53 Unclassified_Arcobacteraceae 0.06

Aquabacterium 0.52 Unclassified_Spirosomaceae 0.06

Peredibacter 0.52 Acidipila-Silvibacterium 0.06

Micrococcus 0.51 Legionella 0.05

Curvibacter 0.48 Ilumatobacter 0.05

Luteimonas 0.47 Kribbella 0.05

Chryseomicrobium 0.47 Rivicola 0.05

Devosia 0.45 Unclassified_Geminicoccaceae 0.05

Allorhizobium-Neorhizobium-Pararhizobium-Rhizobium 0.44 Unclassified_Vicinamibacteraceae 0.05

Acidovorax 0.44 Unclassified_Sandaracinaceae 0.05

Methylophaga 0.43 Gaiella 0.05

Unclassified_Rhodobacteraceae 0.41 Micropruina 0.05

Unclassified_Oxalobacteraceae 0.40 Unclassified_Opitutaceae 0.05

Unclassified_Intrasporangiaceae 0.40 Unclassified_Actinomarinales 0.05

Mesorhizobium 0.38 Bryobacter 0.05

Unclassified_Gemmatimonadaceae 0.37 Longimicrobium 0.05

Cupriavidus 0.37 Noviherbaspirillum 0.05

Phenylobacterium 0.37 Sphingopyxis 0.05

Sphingobium 0.37 Alicyclobacillus 0.04

Thalassospira 0.37 Unclassified_Microbacteriaceae 0.04

Massilia 0.35 Unclassified_Rhizobiales 0.04

Enhydrobacter 0.35 [Eubacterium] ventriosum group 0.04

Ketobacter 0.35 [Ruminococcus] torques group 0.04

Roseomonas 0.35 Psychrobacter 0.04

Aliihoeflea 0.33 Acidiphilium 0.04

Anoxybacillus 0.32 Altererythrobacter 0.04

Jeotgalibacillus 0.32 Aurantisolimonas 0.04

Microbacterium 0.31 Unclassified_Desulfarculaceae 0.04

Amaricoccus 0.30 Unclassified_Phycisphaeraceae 0.04

Alkanindiges 0.29 Unclassified_Subgroup 17 0.04

Citricoccus 0.28 Unclassified_Gaiellales 0.03

Desulfocapsa 0.27 Barnesiella 0.03

Paeniglutamicibacter 0.26 BD1-7 clade 0.03

AAP99 0.25 Cloacibacterium 0.03

Nocardioides 0.25 Haemophilus 0.03

Alishewanella 0.24 OM60(NOR5) clade 0.03

Thauera 0.23 Unclassified_MB-A2-108 0.03

Unclassified_Sh765B-AG-111 0.23 Cyanobium PCC-6307 0.03

Glutamicibacter 0.22 Porphyrobacter 0.03

Maricaulis 0.21 Unclassified_Acetobacteraceae 0.03

Unclassified_Cellulomonadaceae 0.21 Unclassified_WD2101 soil group 0.03

Unclassified_Beijerinckiaceae 0.21 Unclassified_Polyangiaceae 0.03

Brevibacterium 0.21 Unclassified_Bacteroidia 0.03

Unclassified_Xanthomonadaceae 0.21 Gemmata 0.03

Limnobacter 0.20 [Ruminococcus] gnavus group 0.03

Leucobacter 0.20 Hymenobacter 0.03

Sphingobacterium 0.19 Mizugakiibacter 0.03

Ralstonia 0.18 Pajaroellobacter 0.03

Domibacillus 0.18 Pseudogracilibacillus 0.03

possible genus 04 0.18 Unclassified_Latescibacterota 0.03

Gordonia 0.17 WCHB1-32 0.03

Salinicoccus 0.17 Flavisolibacter 0.02

SAR92 clade 0.16 Unclassified_Gemmataceae 0.02

Afipia 0.16 Unclassified_Subgroup 25 0.02

Kocuria 0.16 Candidatus Udaeobacter 0.02

Ochrobactrum 0.15 Solirubrobacter 0.02

Unclassified_JG30-KF-CM45 0.15 Ahniella 0.02

Marinilactibacillus 0.14 Unclassified_Steroidobacteraceae 0.02

Zoogloea 0.14 Flavobacterium 0.01

Lysinibacillus 0.14 Unclassified_Saprospiraceae 0.01

Unclassified_Alphaproteobacteria 0.14 Unclassified_Microtrichales 0.01

Halarcobacter 0.13 Lautropia 0.01

Pseudoxanthomonas 0.13 Nitrosomonas 0.01

Cnuella 0.12 TM7a 0.01

Unclassified_67-14 0.12 Unclassified_Armatimonadota 0.01

Haliangium 0.11 Unclassified_Micromonosporaceae 0.01

Bdellovibrio 0.11 Unclassified_Prevotellaceae 0.01

Chujaibacter 0.11 Unclassified_Roseiflexaceae 0.01

Cutibacterium 0.11 Unclassified_Vicinamibacterales 0.01

Lacunisphaera 0.11 Unclassified_SC-I-84 0.01

Methyloversatilis 0.11 Kouleothrix 0.01

Thiomicrorhabdus 0.11 P3OB-42 0.01

Unclassified_Gitt-GS-136 0.11 Proteiniclasticum 0.01

Unclassified_Isosphaeraceae 0.10 Unclassified_Bacteria 0.01

Xanthomonas 0.10 Unclassified_Micropepsaceae 0.01

Candidatus Competibacter 0.10 Unclassified_PLTA13 0.01

hgcI clade 0.10 Bacteroides 0.01

Unclassified_Rhizobiaceae 0.09 Actinomyces 0.01

Chryseobacterium 0.09 Aquibacter 0.01

Aneurinibacillus 0.09 Syntrophus 0.01

Asticcacaulis 0.09 Unclassified_Hydrogenophilaceae 0.01

Enterococcus 0.09 Unclassified_LD-RB-34 0.01

Sulfuritalea 0.09 Unclassified_mle1-27 0.01

**Table S6. Relative abundance of all bacterial genera in the HY7**

Genus HY7 Genus HY7

Burkholderia-Caballeronia-Paraburkholderia 30.49 Syntrophaceticus 0.31

Brevundimonas 2.68 Unclassified_Subgroup 7 0.30

Unclassified_Microbacteriaceae 1.98 Mesorhizobium 0.30

Pseudoxanthomonas 1.96 Achromobacter 0.29

Pseudomonas 1.90 Azospirillum 0.29

Stenotrophomonas 1.63 Luteitalea 0.28

Flavobacterium 1.39 Methylobacterium-Methylorubrum 0.28

Unclassified_Rhizobiales 1.36 Unclassified_Oxalobacteraceae 0.28

Unclassified_Comamonadaceae 1.36 Actinotalea 0.28

Unclassified_Bacillaceae 1.33 Aeromicrobium 0.28

Unclassified_Rhizobiaceae 1.22 Unclassified_Vicinamibacteraceae 0.27

Terrimonas 1.21 Unclassified_AD3 0.27

Nocardioides 1.17 Unclassified_Gammaproteobacteria 0.26

Unclassified_Enterobacteriaceae 1.15 Pseudorhodoplanes 0.26

Unclassified_Microscillaceae 1.08 Tsukamurella 0.26

Devosia 1.05 Unclassified_Balneolaceae 0.26

Unclassified_A4b 1.03 Shewanella 0.25

Unclassified_Actinomarinales 0.90 Unclassified_Saccharimonadales 0.25

Turneriella 0.83 Cellvibrio 0.24

KD3-10 0.80 Algoriphagus 0.24

Unclassified_S0134 terrestrial group 0.76 Nubsella 0.24

Sphingobacterium 0.74 Unclassified_Proteobacteria 0.24

Pirellula 0.72 Gemmatimonas 0.24

Unclassified_BIrii41 0.72 Kaistia 0.24

Hyphomicrobium 0.72 Pseudopedobacter 0.24

Citrobacter 0.71 Unclassified_Vicinamibacterales 0.23

Corynebacterium 0.69 Ralstonia 0.23

Unclassified_Caldilineaceae 0.69 Paludisphaera 0.23

Undibacterium 0.67 Cutibacterium 0.23

Pseudolabrys 0.67 Renibacterium 0.23

Ochrobactrum 0.66 Tetrasphaera 0.23

Unclassified_Caulobacteraceae 0.63 Unclassified_Gaiellales 0.22

Unclassified_JG30-KF-CM45 0.61 Fenollaria 0.22

Pseudofulvimonas 0.60 Unclassified_Gracilibacteria 0.22

Unclassified_Saprospiraceae 0.59 Silvanigrella 0.21

Unclassified_Alcaligenaceae 0.58 Unclassified_Pirellulaceae 0.21

Unclassified_Pseudomonadales 0.57 Oikopleura 0.21

Unclassified_WD2101 soil group 0.54 Legionella 0.20

Unclassified_Ilumatobacteraceae 0.53 Unclassified_Candidatus Kaiserbacteria 0.20

Bacillus 0.52 Unclassified_Frankiales 0.20

Hydrogenophaga 0.51 Unclassified_Rhodanobacteraceae 0.20

Unclassified_Gemmatimonadaceae 0.51 Unclassified_Blastocatellaceae 0.20

Caulobacter 0.49 Unclassified_Streptomycetaceae 0.20

Unclassified_R7C24 0.49 Unclassified_Geodermatophilaceae 0.19

Phyllobacterium 0.48 Unclassified_Oligoflexaceae 0.19

Unclassified_Hydrogenispora 0.48 Candidatus Udaeobacter 0.19

Lysinibacillus 0.47 Solirubrobacter 0.17

Acinetobacter 0.47 Clostridium sensu stricto 11 0.17

Edaphobaculum 0.46 Unclassified_D05-2 0.16

Sphingomonas 0.45 Unclassified_Microgenomatia 0.15

Mycobacterium 0.45 Dokdonella 0.15

Pseudochrobactrum 0.45 Thermobifida 0.15

Pantoea 0.45 Chryseobacterium 0.15

Unclassified_Diplorickettsiaceae 0.45 Unclassified_SM2D12 0.14

Unclassified_Methylacidiphilaceae 0.44 Unclassified_Subgroup 22 0.14

Virgibacillus 0.44 Halomonas 0.13

Chroococcidiopsis SAG 2023 0.42 Unclassified_Enterobacterales 0.12

Brevibacterium 0.42 Skermanella 0.12

Taibaiella 0.41 Psychrobacter 0.10

Allorhizobium-Neorhizobium-Pararhizobium-Rhizobium 0.38 Unclassified_Sandaracinaceae 0.09

Unclassified_Chitinophagaceae 0.38 Ilumatobacter 0.08

Bosea 0.38 Candidatus Ovatusbacter 0.08

Unclassified_Hyphomicrobiaceae 0.38 Alcaligenes 0.08

Unclassified_Longimicrobiaceae 0.38 Unclassified_Tepidisphaerales 0.08

Unclassified_Burkholderiales 0.38 MND1 0.08

Bryobacter 0.37 Unclassified_SC-I-84 0.07

Bacteroides 0.37 Methylotenera 0.07

Microbacterium 0.36 Unclassified_Anaerolineaceae 0.07

Georgenia 0.35 Sandaracinus 0.06

Unclassified_CCD24 0.35 Hydrogenobacter 0.06

Haliangium 0.35 Unclassified_Mitochondria 0.05

Chitinophaga 0.34 IMCC26207 0.04

Unclassified_Polyangiaceae 0.34 Unclassified_Xanthobacteraceae 0.04

Geobacillus 0.34 Unclassified_Microtrichales 0.04

Luteolibacter 0.34 Hirschia 0.04

Unclassified_Bacteroidia 0.34 Unclassified_LWQ8 0.03

Staphylococcus 0.33 Gemmata 0.02

Aerococcus 0.32 Candidatus Actinomarina 0.01

Unclassified_Micrococcales 0.32 Unclassified_Candidatus Pacebacteria 0.01

Unclassified_Sphingomonadaceae 0.32 Unclassified_Chloroplast 0.01

Pedobacter 0.31

**Table S7. α-diversity calculated from functional gene profile**

Sample Shannon Simpson Chao1

HY1-1 4.23 0.96 291.75

HY7 4.03 0.95 190.37

**Table S8. Relative abundance (base on TPM) of functional genes predicted in KEGG pathway analysis within the microbial community in shale core**

Metablism Gene HY1-1 HY7

Aerobic alkane degradation alkB1_2 0.03 3.57

Aerobic alkane degradation ladA 0.01 4.70

acyl-CoA dehydrogenase E1.3.3.6 0.00 3.12

acyl-CoA dehydrogenase ACADS 0.00 5.27

acyl-CoA dehydrogenase ACADM 0.76 7.34

acyl-CoA dehydrogenase DCAA 0.01 3.30

acyl-CoA dehydrogenase aidB 0.00 1.16

acyl-CoA dehydrogenase acdH 0.01 0.41

long-chain acyl-CoA synthetase ACSL 0.81 6.53

enoyl-CoA hydratase paaF 0.00 3.55

enoyl-CoA hydratase crt 0.00 8.17

enoyl-CoA hydratase fadJ 1 0.50 4.55

enoyl-CoA hydratase ECHS1 0.00 0.37

enoyl-CoA hydratase K15016 1 0.00 3.14

enoyl-CoA hydratase fadB 0.30 0.00

3-hydroxyacyl-CoA dehydrogenase HADH 0.11 2.15

3-hydroxyacyl-CoA dehydrogenase paaH 0.48 7.03

3-hydroxyacyl-CoA dehydrogenase fadJ 2 0.00 4.55

3-hydroxyacyl-CoA dehydrogenase fadN 0.16 6.27

3-hydroxyacyl-CoA dehydrogenase K15016 2 0.00 3.14

acetyl-CoA acyltransferase ACAT 0.00 7.84

acetyl-CoA acyltransferase fadA 0.41 6.18

acetyl-CoA acyltransferase E2.3.1.9 0.32 0.00

Coenzyme M synthesis com3 0.00 6.33

Coenzyme M synthesis com2 0.00 6.41

Coenzyme M synthesis com5 0.00 2.13

Coenzyme M synthesis com1 0.00 6.17

Coenzyme M synthesis com4 0.00 1.82

Methylotrophic methanogenesis mer 0.00 6.37

Methylotrophic methanogenesis mttB 0.00 0.97

Methylotrophic methanogenesis mtsB 0.00 4.19

Hydrogenotrophic methanogenesis mtrA 0.00 6.00

Aerobic Benzoate degradation fadA 0.41 6.18

Aerobic Benzoate degradation pcaD 0.19 5.77

Aerobic Benzoate degradation pcaC 0.00 5.60

Aerobic Benzoate degradation pcaB 0.03 0.70

Aerobic Benzoate degradation catA 0.02 6.15

Aerobic Benzoate degradation catC 0.00 0.94

Aerobic Benzoate degradation benC-xylZ 0.02 7.32

Aerobic Benzoate degradation pcaF 0.13 4.62

Acetate metabolism ackA 0.41 0.54

Acetate metabolism acyP 0.01 4.87

Acetate metabolism ACSS1_2 0.00 7.55

Acetate metabolism acdAB 0.00 2.10

Acetate metabolism ACSS 0.44 0.00

Propanoate degradation SDHD 0.00 3.95

Metablism Gene HY1-1 HY7

Propanoate degradation sdhA 0.11 7.46

Propanoate degradation sdhB 0.09 5.61

Propanoate degradation sdhC 0.10 4.46

Propanoate degradation sdhD 0.02 4.53

Propanoate degradation MUT 0.29 5.23

Propanoate degradation E5.4.99.2A 0.00 6.76

Propanoate degradation E5.4.99.2B 0.00 6.45

Propanoate degradation sucD 0.29 6.74

Propanoate degradation sucC 0.29 6.93

Propanoate degradation PCCA 0.02 1.14

Propanoate degradation PCCB 0.02 7.30

Propanoate degradation MCEE 0.00 7.33

Propanoate degradation bccA 0.01 1.49

Propanoate Transporter actP 0.13 0.90

Nitrate reduction narJ 0.00 1.89

Nitrate reduction nrtA 0.08 3.80

Nitrate reduction nrtB 0.07 3.80

Nitrate reduction nrtD 0.00 1.20

Nitric oxide reductase norB 0.28 5.61

Nitrification (comammox) pmoA-amoA 0.00 5.95

Nitrification (comammox) pmoB-amoB 0.00 5.61

Nitrification (comammox) pmoC-amoC 0.00 7.20

Nitrite reductase nirK 0.12 7.71

Nitrite reductase nirB 0.27 4.97

Nitrite reductase nirD 0.00 3.95

Nitrite reductase nirS 0.18 0.00

Nitrous-oxide reductase nosZ 0.08 0.00

Disimilatory sulfate reducing dmsB 0.00 4.92

Disimilatory sulfate reducing dmsC 0.00 4.80

Disimilatory sulfate reducing sat 0.00 7.14

Sulfur reduction sqr 0.01 8.86

aerobic carbon-monoxide dehydrogenase coxS 0.00 3.75

aerobic carbon-monoxide dehydrogenase coxM, cutM 0.00 4.50

aerobic carbon-monoxide dehydrogenase coxL, cutL 0.00 3.59

Glycolysis gap2 0.00 6.85

Glycolysis porA 0.00 5.56

Glycolysis porB 0.00 5.78

Glycolysis porD 0.00 4.57

Glycolysis korA 0.03 7.36

Glycolysis korB 0.00 6.75

Glycolysis glk 0.09 5.06

Glycolysis pfkA 0.00 4.34

Glycolysis PK 0.43 7.06

Glycolysis K01622 2 0.00 6.59

Glycolysis ALDO 0.07 1.38

Glycolysis FBA 0.04 3.32

Metablism Gene HY1-1 HY7

Glycolysis ENO1_2_3 0.00 5.97

Glycolysis GPI 0.58 5.11

Glycolysis glpX 0.01 1.46

Glycolysis por 0.65 0.56

Glycolysis FBP 0.04 5.78

Glycolysis pgi1 0.00 0.80

Glycolysis fbaB 0.09 4.03

Glycolysis tal-pgi 0.00 2.23

Glycolysis gpmI 0.09 6.17

Glycolysis gpmB 0.00 7.13

Glycolysis apgM 0.00 6.84

Glycolysis pgi-pmi 0.00 5.96

Glycolysis K16306 0.00 2.76

Glycolysis pfkB 2.35 2.06

Glycolysis ENO 0.29 0.00

Glycolysis fbp3 0.03 0.00


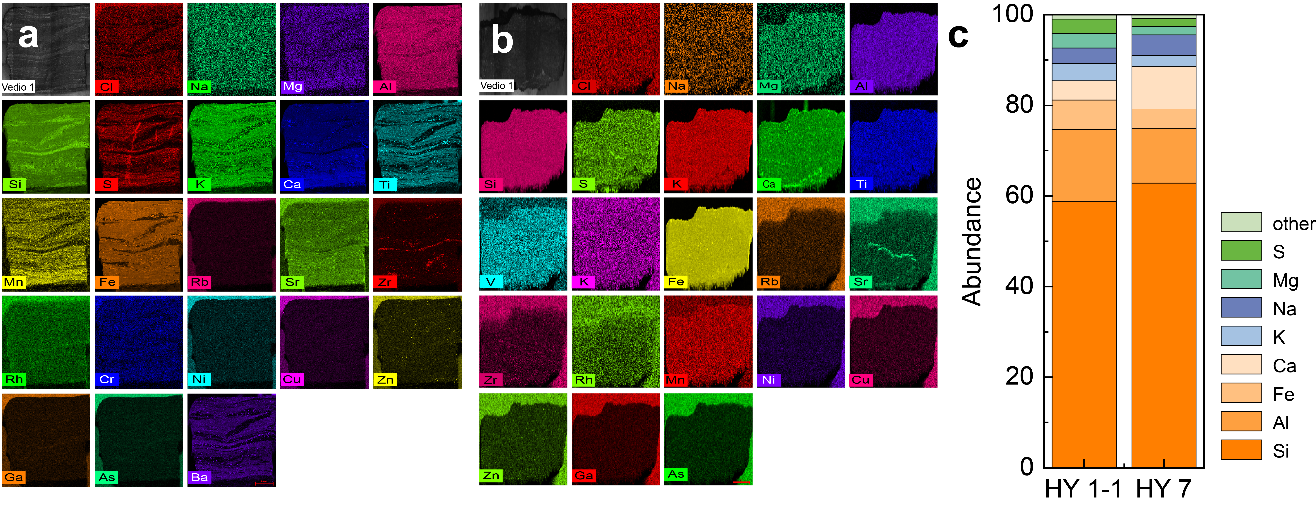


**Figure S1. Elemental mapping of HY1-1 (a) and HY7 (b) detected by X-ray Fluorescence spectra.**

**
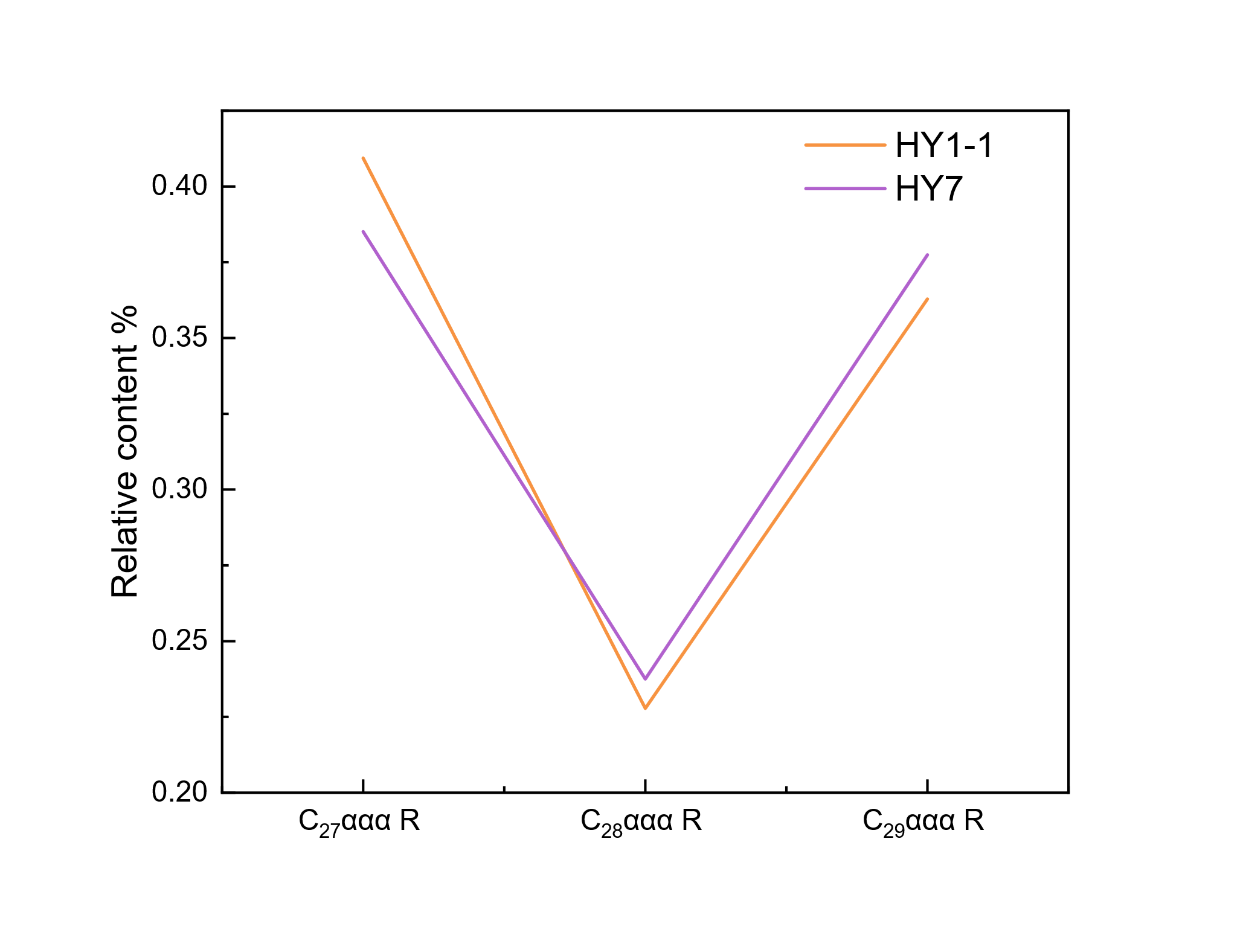
**

**Figure S2. The relative distribution of C27αααR, C28αααR, and C29αααR steranes indicates dual contributions from both aquatic organisms and higher plants.**


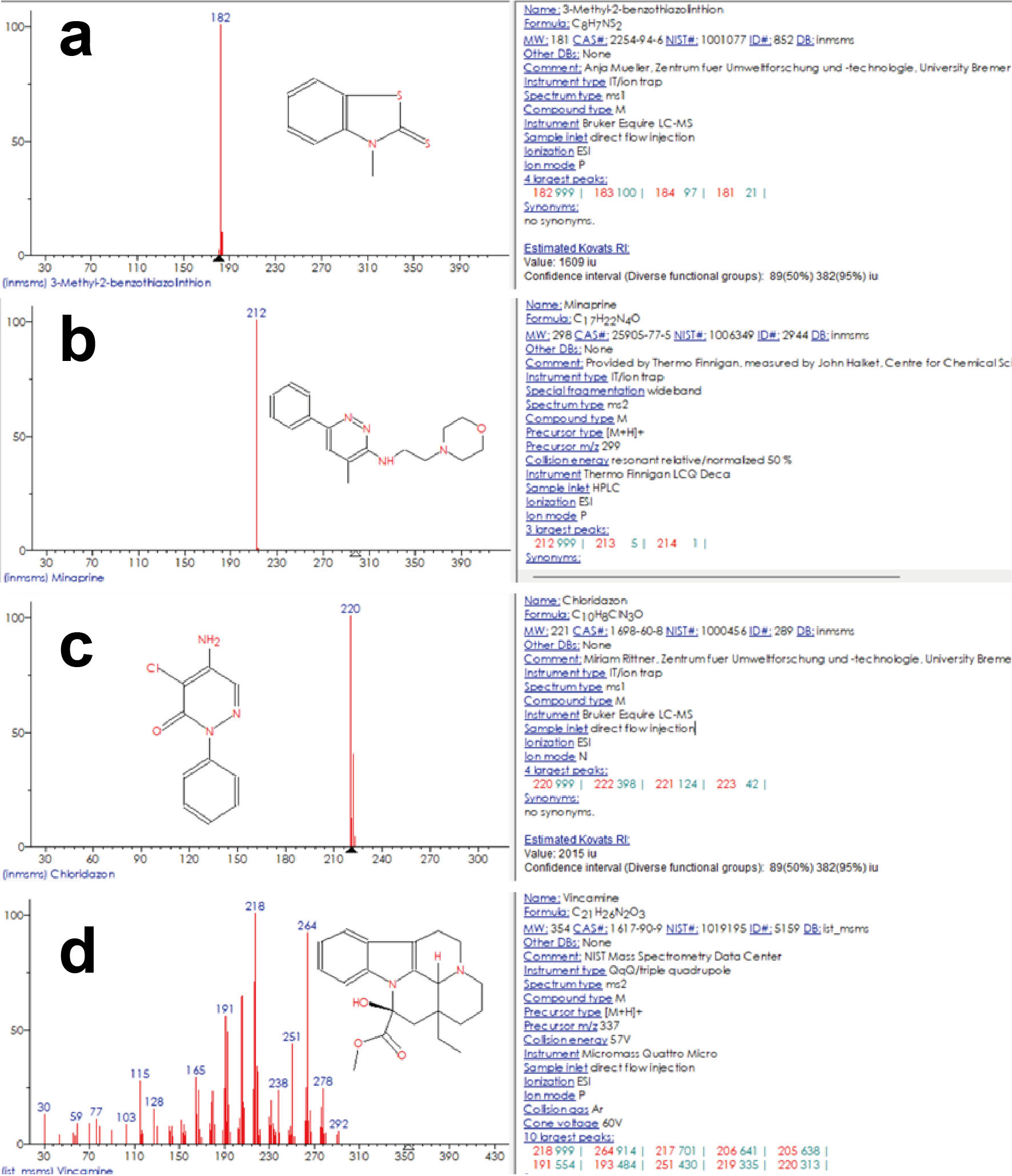


**Figure S3. The nitrogen-containing compounds 3-Methyl-2-benzothiazolinthion (a), Minaprine (b), Chloridazon (c), and Vincamine (d) identified in the aromatic hydrocarbon fractions using GC-MS.**


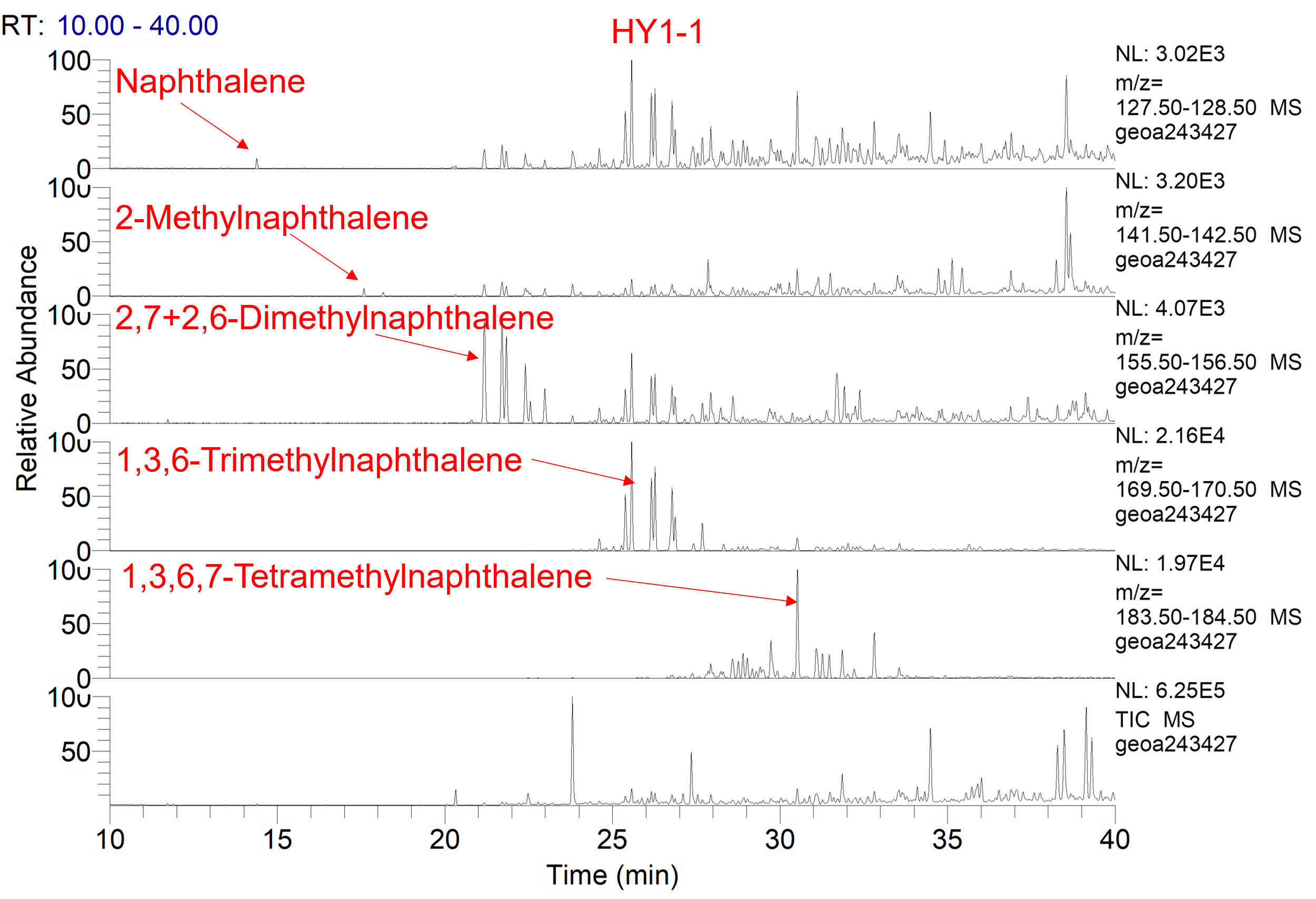

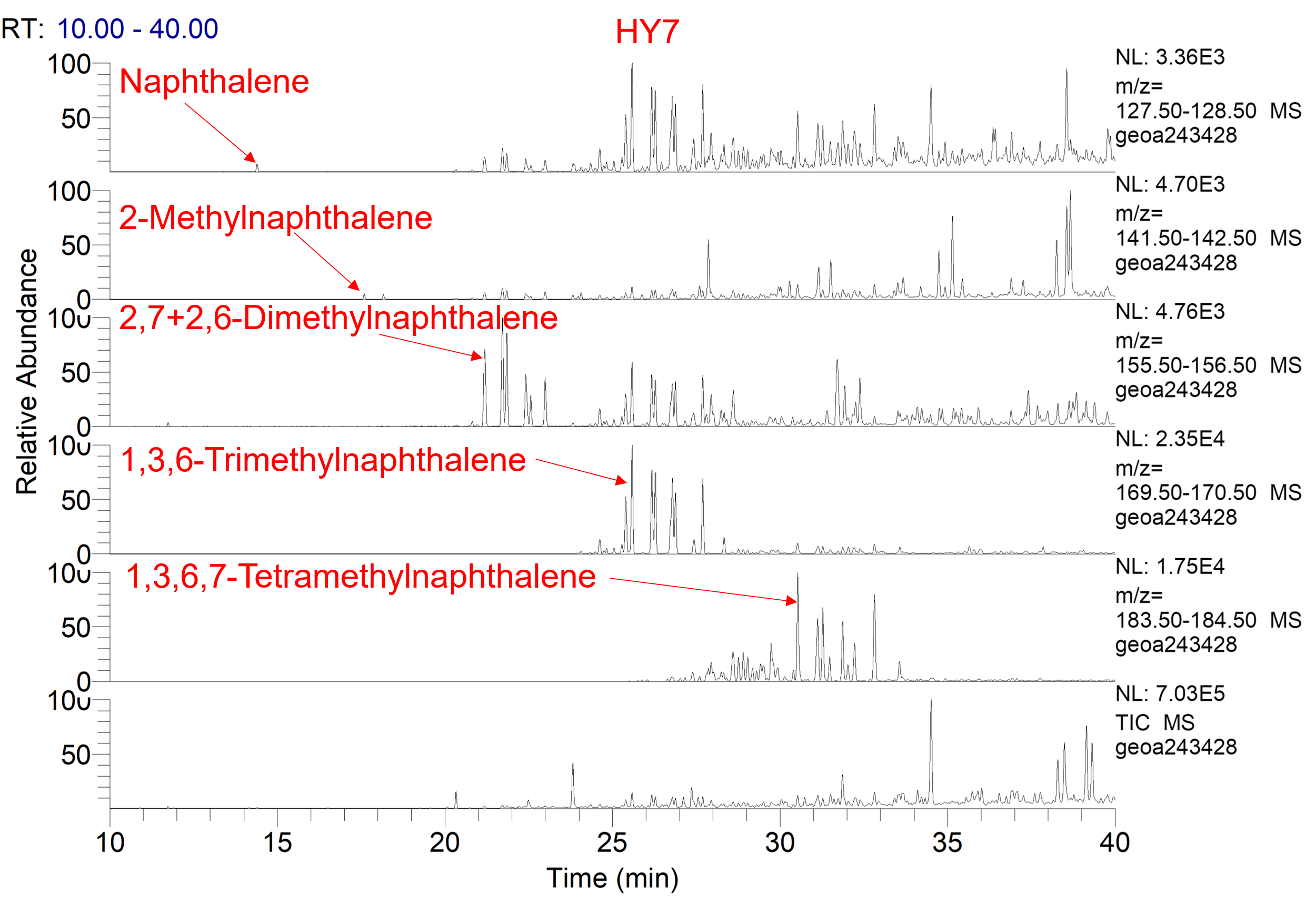


**Figure S4. Representative aromatic compounds identified in the samples.**
